# Exploiting loss of heterozygosity for allele-selective colorectal cancer chemotherapy

**DOI:** 10.1038/s41467-020-15111-4

**Published:** 2020-03-11

**Authors:** Veronica Rendo, Ivaylo Stoimenov, André Mateus, Elin Sjöberg, Richard Svensson, Anna-Lena Gustavsson, Lars Johansson, Adrian Ng, Casey OʼBrien, Marios Giannakis, Per Artursson, Peter Nygren, Ian Cheong, Tobias Sjöblom

**Affiliations:** 1 Science for Life Laboratory, Department of Immunology, Genetics and Pathology, Uppsala University, SE-75185 Uppsala, Sweden; 2 Dana-Farber Cancer Institute, 450 Brookline Ave, 02115 Boston, MA USA; 3 Uppsala Drug Optimization and Pharmaceutical Profiling Facility (UDOPP), SciLifeLab Chemical Biology Consortium Sweden (CBCS), Department of Pharmacy, Uppsala University, 75123 Uppsala, Sweden; 4 SciLifeLab Drug Discovery and Development Platform, ADME of Therapeutics facility (UDOPP), Department of Pharmacy, Uppsala University, 75123 Uppsala, Sweden; 5 Chemical Biology Consortium Sweden, Science for Life Laboratory, Division of Translational Medicine and Chemical Biology, Department of Medicinal Biochemistry and Biophysics, Karolinska Institute, 17177 Solna, Sweden; 6 Temasek Life Sciences Laboratory, 1 Research Link, NUS, 117604 Singapore, Singapore

**Keywords:** Targeted therapies, Colon cancer, Phenotypic screening

## Abstract

Cancer chemotherapy targeting frequent loss of heterozygosity events is an attractive concept, since tumor cells may lack enzymatic activities present in normal constitutional cells. To find exploitable targets, we map prevalent genetic polymorphisms to protein structures and identify 45 nsSNVs (non-synonymous small nucleotide variations) near the catalytic sites of 17 enzymes frequently lost in cancer. For proof of concept, we select the gastrointestinal drug metabolic enzyme *NAT2* at 8p22, which is frequently lost in colorectal cancers and has a common variant with 10-fold reduced activity. Small molecule screening results in a cytotoxic kinase inhibitor that impairs growth of cells with slow NAT2 and decreases the growth of tumors with slow NAT2 by half as compared to those with wild-type NAT2. Most of the patient-derived CRC cells expressing slow NAT2 also show sensitivity to 6-(4-aminophenyl)-N-(3,4,5-trimethoxyphenyl)pyrazin-2-amine (APA) treatment. These findings indicate that the therapeutic index of anti-cancer drugs can be altered by bystander mutations affecting drug metabolic genes.

## Abstract (web summary)

Allelic losses occurring in cancer cells have been suggested as potential targets for therapy. Here, the authors show how recurring loss of heterozygosity of a drug metabolic gene in colorectal cancers can be exploited using a low molecular weight compound.

## Introduction

Recent targeted anti-cancer therapies exploit acquired genetic differences between cancer and normal cells, such as activation by mutation of a specific oncogene, inactivation by mutation of a tumor suppressor gene, or perturbation of pathways involved in the maintenance of genome integrity to achieve preferential killing of cancer cells^1^. Drugs targeting protein tyrosine kinases are mainstays of clinical cancer care^2–4^, and strategies addressing loss-of-function of tumor suppressor genes such as *TP53*^5,6^ and *RB*^7^ are in development. Collateral lethality, exploiting vulnerabilities left in cancer cells after a passenger gene in the vicinity of a tumor suppressor is lost, has emerged as a new therapeutic avenue. The frequent 1p36 deletion in glioblastoma, entailing loss of *ENO1*, sensitizes tumor cells to ENO2 inhibition^8^. Similarly, complete losses of *POLR2A*, *MTAP*, and *ME3*^9–12^, and partial losses of *PSMC2*^13^, *SF3B1*^14^, and *MAGOH*^15^ have been identified as potential therapeutic targets in human cancers. Targeting of the loss of heterozygosity (LOH) events occurring as cancer genomes inactivate tumor suppressor genes has been achieved by allele-specific inhibition, where variants of essential genes such as the 70-kDa subunit of replication protein A (*RPA70*) near *TP53* are silenced using antisense oligonucleotides^16^. However, allele-specific LOH therapy targeting proteins tractable to inhibition by systemically administered agents has previously not been demonstrated. We here demonstrate that a recurring loss of heterozygosity event affecting a drug metabolic activity (*NAT2*) can increase the sensitivity to a low molecular weight cytotoxic compound.

## Results

### Identification of N-acetyltransferase 2 (NAT2) as a target for allele-specific inhibition

**Fig. 1:**
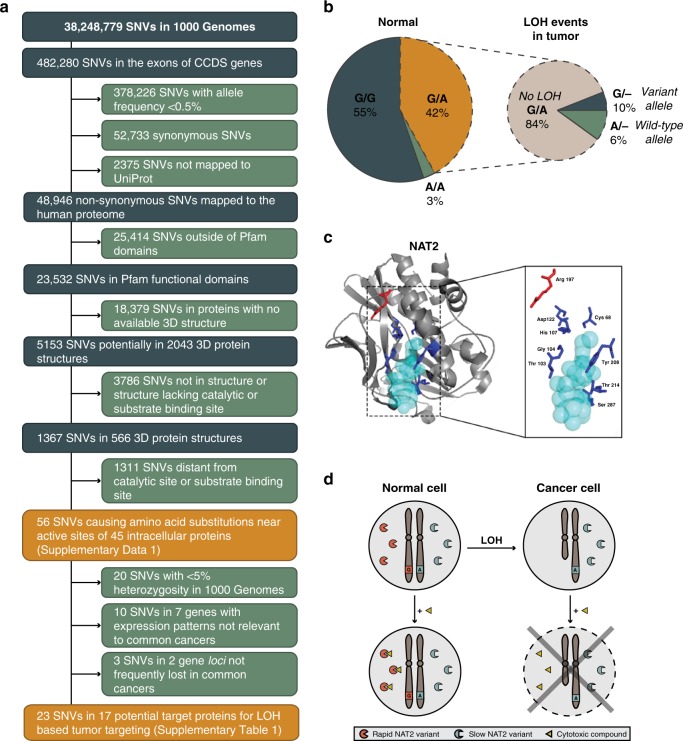
N-acetyltransferase 2 (NAT2) is a target for allele-specific inhibition. **a** Identification of enzymes with prevalent alternative alleles resulting in amino-acid substitution near functional sites. A series of filtering steps was applied to identify the subset of SNVs causing amino-acid substitutions near active sites in publicly available 3D protein structures. To enrich potential target proteins for LOH-based tumor targeting approaches, the candidate genes were further selected based on expression profile and the frequency of LOH in common cancer types. **b** Loss of heterozygosity at 8p22 can render CRCs deficient in NAT2 function. To confirm the prevalence of rs1799930 and the frequency of LOH at NAT2, genomic DNA from 74 CIN CRCs and patient-matched normal tissues was genotyped. Left, genotype distribution of normal tissues. Right, LOH events observed in the tumors of heterozygous individuals. **c** Structure of human NAT2 co-crystallized with the cofactor coenzyme A (light blue) (PDB: 2PFR). The side chains of the active site amino-acid residues involved in substrate transformation (Asp122, Cys68, and His107) and cofactor binding (Gly104, Thr103, Thr214, Tyr208, and Ser287) (dark blue) and the side chain of Arg197 (rs1799930, red) are shown. The figure was designed in PyMOL (v. 2.3.2). **d** Exploiting loss of a rapid NAT2 allele in tumor cells for anti-cancer therapy. Eligible patients are heterozygous for the slow (A, blue) and rapid (G, red) NAT2 alleles. During cancer progression, cancer cells may undergo loss of heterozygosity (LOH) and lose the rapid NAT2 allele (red). Treatment with a cytotoxic compound (triangle) that can only be processed by the rapid NAT2 enzymatic variant lost in cancer cells will result in selective tumor death.

To identify target proteins, we mapped variants observed in the 1092 individuals in the 1000 Genomes project to functional domains and crystal structures to identify those that could alter protein structure in catalytic or substrate binding sites (Fig. 1a). The variants were first mapped to transcripts, localizing 482,280 small nucleotide variants (SNVs) to protein coding regions. Of these, 78% were likely to be present in only one population^17^. To enrich for prevalent targets of higher utility in LOH-directed therapies, 23,532 non-synonymous SNVs in functional protein domains with allele frequency ≥0.5% were selected. Of these, 1367 SNVs (~5.8%) mapping to 566 crystal structures had both the SNV and active or substrate binding sites defined in the structure. After visual inspection, 56 SNVs in 45 intracellular proteins resulted in amino-acid substitutions near catalytic residues or substrate binding pockets, including 36 SNVs in 26 genes with >5% heterozygosity in all 1000 Genomes populations (Supplementary Data 1). Finally, retaining only the genes with >15% LOH frequency in common human cancers^18^ and with a gene expression profile matching the tissues of interest, yielded 17 potential target enzymes for LOH-directed cancer therapies (Supplementary Table 1). From the putative target genes, we selected polymorphisms in *NAT2*, *AKR7A2*, and *SULT1A1* for validation as these genes were known to be involved in drug metabolism and are non-essential for cell survival. Other genes with common variants were considered but not prioritized because of likely limited chemical space of substrates (*HSD17B*), small reduction in catalytic activity by the variant (*GSTP1*), unknown importance (*HAAO*), known substrate redundancy with related enzymes (*GSTP1*, *SULT*s), or too few LOH events in CRC (*ABP1* and *AKR7A*). To confirm the expected prevalence of candidate SNVs and frequency of LOH events, we genotyped the selected SNVs in 74 patients with chromosomally unstable colorectal cancers (CRCs) and could detect all in heterozygous states (Supplementary Table 2). Next, tumors from heterozygous individuals were assessed for somatic LOH events. The highest frequency of allelic loss was observed for *NAT2*, where ~7% heterozygous for the rs1799930 polymorphism retained only one allele in their tumors because of LOH (Fig. 1b; Supplementary Table 2). We observed a similar likelihood for CRCs to lose either allele (Fig. 1b), in an LOH event detected in stage II as well as stage III and IV CRCs (Supplementary Fig. 1), in line with *NAT2* being a bystander gene on 8p which is lost early in CRC development^19^. After genotyping, based on the frequency of the LOH events *NAT2* was prioritized as a proof of concept gene over *AKR7A2* and *SULT1A1*. The *NAT2* gene encodes one of the two human N-acetyltransferases involved in xenobiotic metabolism of arylamines and hydrazine compounds. *NAT2* is highly polymorphic with 108 allelic variants identified in human populations [http://nat.mbg.duth.gr/]. The SNV rs1799930 defines the NAT2*6 group of variant alleles (R197Q) with ≥10-fold reduced activity compared with wild-type *NAT2*, considered to encode a rapid acetylator phenotype^20,21^ (Fig. 1c). Whereas NAT1 is expressed in essentially all tissues, NAT2 is confined to the liver and gastrointestinal tract (GI)^22,23^. From the putative target proteins identified, we selected NAT2 because of its (a) known role in drug metabolism, (b) non-redundant and well defined substrate specificity, (c) GI restricted expression, (d) location on a chromosome arm frequently lost during transition from colorectal adenoma to carcinoma^24^, (e) 10-fold activity difference between the gene products encoded by the wild-type and a common variant allele, and (f) clinically relevant substrates as ~10 commonly used drugs are subject to NAT2 metabolism, among them the cytotoxic drug amonafide^25^, where rapid acetylators have ~4.5-fold higher plasma ratio of N-acetyl-amonafide to amonafide than slow acetylators leading to increased systemic toxicity^26^.

### Discovery of compounds selectively toxic to slow NAT2 cells

**Fig. 2:**
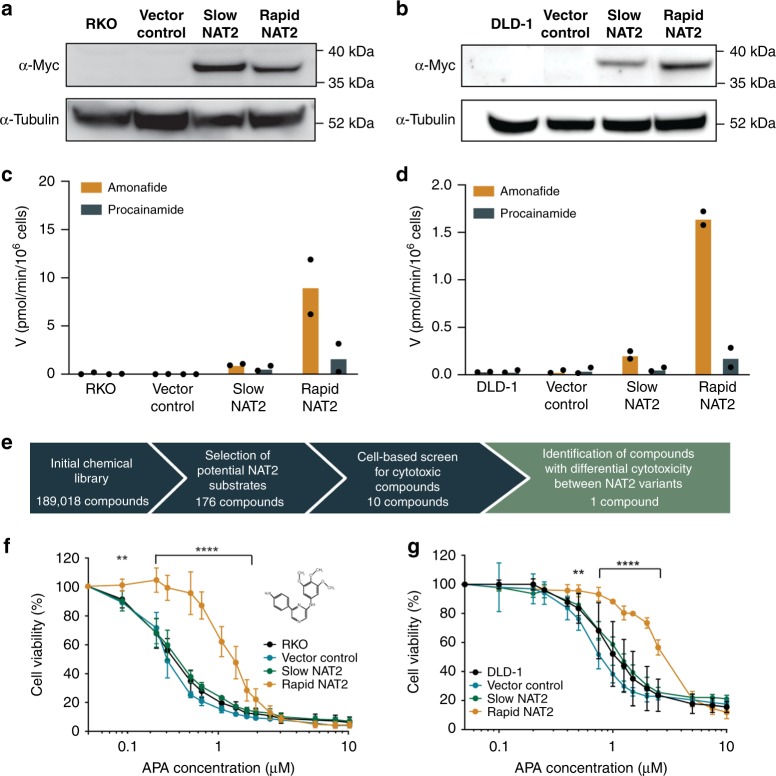
Identification of 6-(4-aminophenyl)-N-(3,4,5-trimethoxyphenyl)pyrazin-2-amine (APA), a compound selectively toxic to slow NAT2 cells. **a**, **b** Validation of RKO (**a**) and DLD-1 (**b**) CRC cell models of rapid and slow NAT2. Cell lysates from clones and parental cells were subjected to PAGE and immunoblotting with an α-MYC-Tag antibody to detect recombinant NAT2 and α-tubulin detection as loading control. **c**, **d** Quantification of NAT2 catalytic activity in RKO and DLD-1 cell clones. The velocity at which the NAT2-specific substrates amonafide (yellow) and procainamide (blue) become acetylated by different enzymatic variants at 10 µM was measured by LC-MS/MS in RKO (**c**) and DLD-1 (**d**) clones. Mean of two independent experiments. **e** Workflow of the selection of the chemical compounds for screening. **f**, **g** The dose-response for APA is NAT2 dependent in both RKO (**f**) and DLD-1 (**g**) CRC cells. Rapid or slow acetylator NAT2 clones, parental cells and vector controls were treated with different concentrations of APA and cell viability was measured by MTT assay after 72 h. The mean and S.D. of three independent experiments is shown. Data were analyzed using two-way ANOVA. **P = 0.0035 in (**f**) and 0.0074 in (**g**), ****P < 0.0001.

**Fig. 3:**
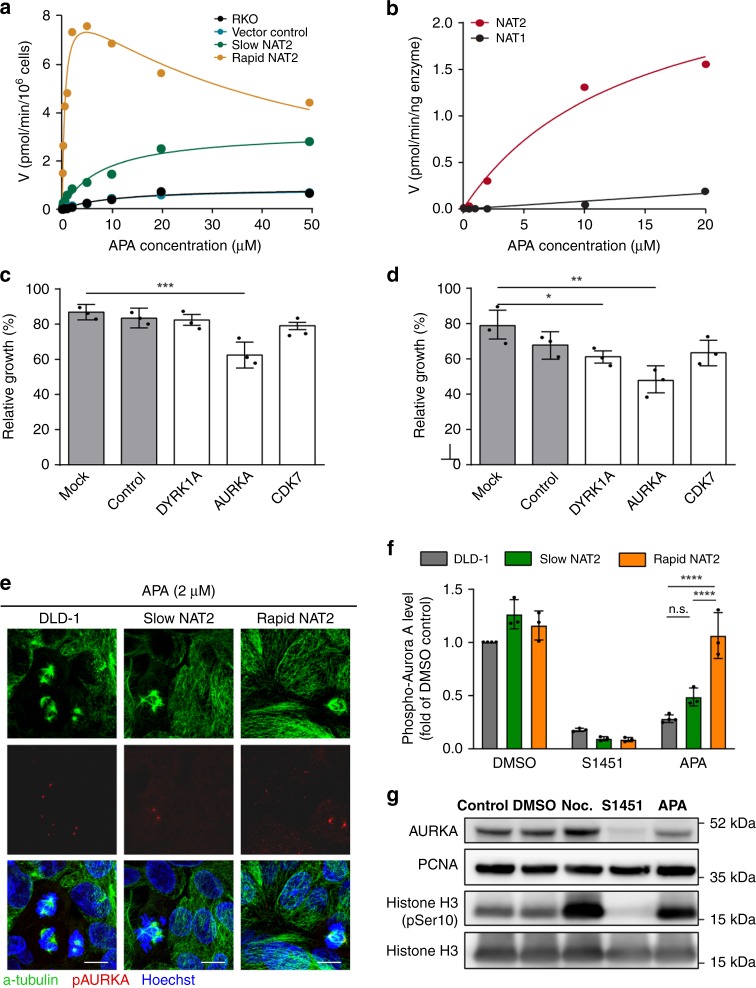
The NAT2-specific substrate APA causes mitotic arrest in slow NAT2 cells. **a** NAT2 mediates APA acetylation. The velocity of NAPA formation was determined by LC-MS/MS in slow (green), rapid (yellow) NAT2 clones, vector control (blue), and parental RKO cells (black) after incubation with APA at indicated concentrations. **b** APA is selectively metabolized by NAT2. Enzyme kinetics of NAPA formation by human recombinant NAT1 (black) and NAT2 (red) proteins. **c**, **d** siRNA-mediated gene silencing of protein kinases with differential inhibition by APA and NAPA expressed in RKO CRC cells. Relative growth of RKO (**c**) and DLD-1 (**d**) cells transfected with a pool of siRNA targeting *DYRK1A*, *AURKA*, *CDK7*, non-target RNA control and mock transfection as percentage of the growth of untransfected cells. Mean and S.D. (error bars) of three independent experiments is shown. Data were analyzed by one-way ANOVA. *P = 0.0435, **P = 0.0013, ***P = 0.0004. **e** APA (2 µM) reduces the phospho-Thr288 Aurora kinase A (pAURKA) staining in NAT2-deficient cells but not in rapid NAT2 clones. Mitotic spindles were visualized by α-tubulin staining (green) and pAURKA levels (red). Representative images of three independent repeats are shown for each cell type. Scale bar, 10 µm. **f** Quantification of pAURKA signal in parental DLD-1 and slow and rapid NAT2 clones after APA treatment (2 µM). The pAURKA levels are shown as a fold of the DMSO control group. Mean and S.D. (error bars) of three independent experiments. Data were analyzed using two-way ANOVA. n.s., *P* = 0.0619 and ****P < 0.0001. **g** APA (2 µM), similarly to the AURKA selective inhibitor S1451 (10 µM) reduces the total protein level of AURKA in contrast to nocodazole (100 ng mL^−1^). DLD-1 cells in different treatments were subjected to immunoblot analysis with different antibodies for detection of total AURKA, PCNA (loading control), histone H3 pSer10, and total histone H3. Representative immunoblot from at least two independent experiments is shown.

We reasoned that anti-cancer drugs rendered less toxic by NAT2 metabolism are also likely to exist and that CRC cells having lost a rapid *NAT2* allele through LOH could be sensitized to treatment with a cytotoxic NAT2 substrate relative to other constitutional cells retaining the rapid allele (Fig. 1d). Therefore, cell systems for small molecule library screening were engineered in human CRC RKO and DLD-1 cells by transfection with NAT2 expression vectors encoding slow NAT2*6A (rs1799930) or rapid NAT2*13A (wt) alleles (Figs. 2a, b). Both cell lines are homozygous for the slow NAT2*6A allele and have low endogenous *NAT2* expression. The acetylation velocities of the NAT2 substrates amonafide and procainamide were ≥8-fold higher in rapid NAT2 clones when compared with slow NAT2 in RKO (*p* < 0.0001) and DLD-1 (*p* < 0.05) clones (Figs. 2c, d). To identify agents with selective toxicity toward CRC cells only expressing slow NAT2, we searched a chemical library for arylamine compounds with demonstrated cytostatic or cytotoxic effects, resulting in 176 candidate substances from a total of 189,018 (Fig. 2e). Next, dose-response experiments were performed to determine cytotoxicity of these potential NAT2 substrate compounds in RKO cells (Supplementary Fig. 2 and Supplementary Table 3). Ten substances showed cytotoxicity at 10 µM and one (6-(4-aminophenyl)-N-(3,4,5-trimethoxyphenyl)pyrazin-2-amine; APA) selectively killed slow NAT2 cells (Fig. 2e). A ~3-fold difference in growth inhibition of the rapid NAT2 clones (EC_50_ 0.97 µM for RKO and 2.94 µM for DLD-1 cells) versus NAT2-deficient cells (EC_50_ 0.33 µM for RKO and 1.21 µM for DLD-1) was detected following APA treatment (Figs. 2f, g). Silencing of endogenous *NAT2* in HCT116 CRC cells harboring rapid and slow *NAT2* alleles sensitized them to APA, but not to 5-fluorouracil (Supplementary Fig. 3). To translate the observed difference in cytotoxicity to NAT2 efficiency, cell-based enzyme kinetics were determined. The rapid NAT2 RKO cells had increased affinity of APA acetylation, and the specificity of rapid versus slow NAT2 (*V*_max_ *K*_m_^−1^) was 40-fold at similar levels of NAT2 protein (Figs. 2a and 3a). Recombinant assays with human NAT1 and NAT2 further proved APA acetylation to be specifically mediated by NAT2 (*V*_max_ *K*_m_^−1^ > 20-fold) (Fig. 3b). Together, APA is a cytotoxic agent which is directly inactivated by NAT2.

### Mediators of APA toxicity in slow NAT2 cells

Structural similarities between APA and kinase inhibitors^27^ prompted investigation of protein kinases as mediators of APA toxicity. We reasoned that N-acetyl-APA (NAPA), the less toxic NAT2 metabolite of APA, should not effectively inhibit the target kinases. When the binding affinity of APA and NAPA to 468 protein kinases was determined, APA had ≥10% binding affinity to 235 human kinases, while NAPA showed reduced binding affinity to 89 of these (Supplementary Fig. 4A). To enrich for kinase interactions that could explain the preferential killing of slow NAT2 cells, we compared the binding affinity of each kinase with APA and NAPA, selected 44 targets with preferential binding to APA and excluded kinases non-essential for cell survival and those not expressed in CRC cells (Supplementary Fig. 4A, Supplementary Table 4). The remaining kinases DYRK1A, AURKA, and CDK7, were subject to siRNA knockdown in RKO and DLD-1 cells (Supplementary Fig. 5A, B) and knockdown of AURKA (Aurora kinase A) resulted in growth inhibition of both (Figs. 3c, d). The growth inhibition of RKO and DLD-1 following siRNA-mediated *AURKA* knockdown (Figs. 3c, d) reflected the relative efficiency of protein downregulation (Supplementary Fig. 5C, D). While APA bound to AURKA (*K*_d_ = 13.5 µM), NAPA did not (Supplementary Fig. 4B). Thermal shift assay demonstrated a thermal unfolding profile of AURKA in presence of APA similar to the AURKA inhibitor Tripolin A^28^, albeit weaker than that of S1451^29^ (Supplementary Fig. 4C). AURKA regulates cell cycle progression through association with other centrosome and spindle-associated proteins. Its activation requires auto-phosphorylation at Thr288 and ensures spindle pole integrity, centrosome duplication as well as adequate chromosomal alignment and segregation^30^, while its inhibition leads to multipolar spindles with acentrosomal poles^28,31^. Upon APA treatment, DLD-1 cells lacking NAT2 had >40% reduced phosphorylation of AURKA at Thr288 (pAURKA), while rapid NAT2 cells did not differ from DMSO-treated control (Figs. 3e, f and Supplementary Fig. 6A). S1451 reduced the fraction of pAURKA levels by >90% regardless of NAT2 expression (Fig. 3f and Supplementary Fig. 6A). Total AURKA protein levels were decreased after APA or S1451 treatment (Fig. 3g)^32^. Treatment with APA increased the levels of the mitotic marker histone H3 pSer10 (Fig. 3g). Further, both APA and nocodazole arrested NAT2-deficient cells in mitosis, as demonstrated by high levels of histone H3 pSer10, whereas only nocodazole arrested NAT2-proficient cells (Supplementary Fig. 6B). The number of cells with mitotic spindles after APA treatment increased by ~15% in DLD-1 cells lacking NAT2, but not in rapid NAT2 cells where <5% cells had mitotic spindles (Supplementary Figs. 6B, C). While the full compendium of mechanisms for APA cytotoxicity remains to be characterized, these results show that AURKA is a target of APA but not NAPA in vitro, and that the cytotoxicity resulting from kinase inhibition may be mediated by AURKA inhibition.

### Evaluation of the in vivo anti-tumor activity of APA

**Fig. 4:**
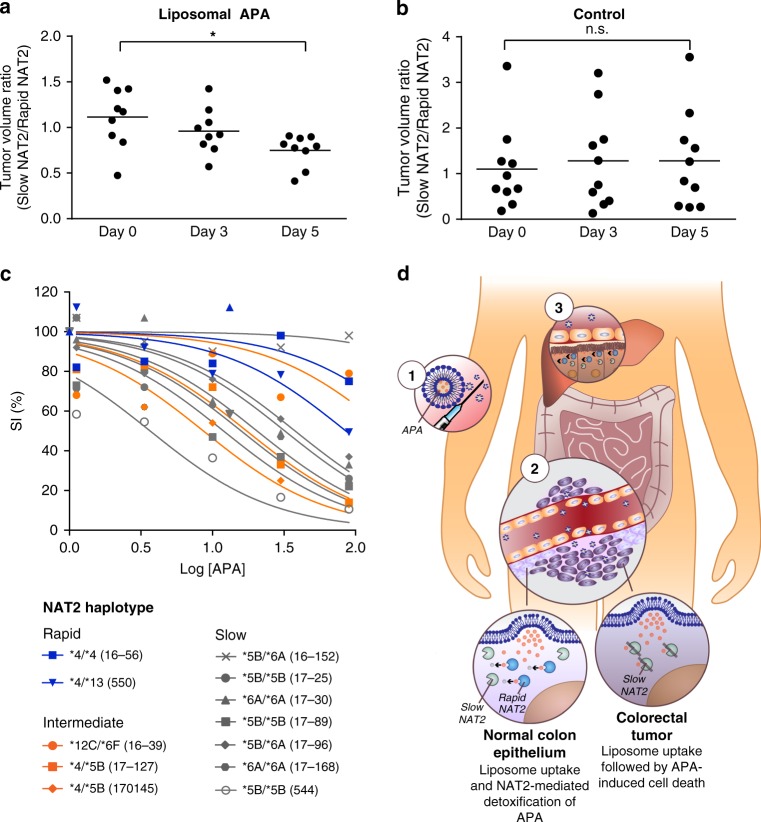
APA shows anti-tumor activity in vivo and is a proof-of-concept compound for colorectal cancer chemotherapy. **a**, **b** Treatment with liposomal APA selectively inhibits growth of slow NAT2 tumors in vivo. Athymic nude mice carrying subcutaneous slow (left flank) and rapid (right flank) NAT2 RKO cell clones received intravenous injections of liposomal APA on days 0 and 3. Results are shown for one representative experiment conducted in 9 mice for the treatment group and 10 mice for the control group. Data were analyzed using one-way ANOVA, n.s., *P* = 0.9013 and *P = 0.0220. **c** Primary CRC tumors show sensitivity toward APA treatment. The survival index (SI) of primary tumor cells and organoids from 12 different CRC patients was determined when exposed to increasing concentrations of APA (log of concentration in μM). Samples encoding rapid (blue), intermediate (orange), and slow (gray) acetylator phenotypes are shown with their respective NAT2 haplotype. **d** A conceptual way of exploiting *NAT2* loss of heterozygosity. Anti-cancer drugs benefit from reduced toxicity and improved tumor uptake when encapsulated in stealth liposomes. First, liposomal APA is injected intravenously in a rapid/slow NAT2 patient whose tumor has lost the rapid allele^1^. The stealth liposomes extravasate through the leaky tumor vessels and accumulate in tumor tissues due to the enhanced permeability and retention (EPR) effect^2^. The lipid bilayer of the liposomes fuses with cell membranes and deliver APA to the colorectal tumor cells and adjacent normal epithelial cells. The adjacent normal epithelial cells express rapid NAT2 and are able to process APA into its nontoxic product NAPA. In contrast, cancer cells expressing only slow NAT2 are not capable of detoxifying APA and die. Liposomal drug formulations also accumulate in the liver, where APA is cleared by rapid NAT2^3^. Every element of (**d**) was created in Adobe Illustrator 2019.

Systemic administration of APA would likely result in limited tumor exposure, as the compound was prone to rapid oxidative metabolism in liver microsomes (Supplementary Table 5). We therefore used sterically-stabilized liposomes to increase tumor targeting via the enhanced perfusion and retention (EPR) effect, protecting normal cells from the toxic substrate and enriching the drug in the tumor^33^. To determine the anti-tumor efficacy of APA, athymic mice xenografted on each flank with rapid and slow NAT2 tumors received free APA, liposomal APA, or control liposomes. Slow NAT2 tumors treated by liposomal or free APA were 50% smaller compared with those treated with control liposomes (*p* < 0.05, Supplementary Fig. 7A). This difference was not observed with rapid NAT2 tumors (Supplementary Fig. 7B). Further, a modest, but measurable >30% decrease in the ratio of slow to rapid NAT2 tumor volumes was observed for animals treated with liposomal APA (*p* < 0.05, Fig. 4a), meaning that rapid NAT2 tumors on average grew faster than the corresponding slow NAT2 tumor on each animal. In comparison, the same ratio was unchanged in mice treated with control liposomes and free drug (Fig. 4b; Supplementary Fig. 7C). The growth inhibitory effect of liposomal but not free APA supports the idea that encapsulation minimizes APA metabolism en route to the tumor by mouse Nat enzymes.

To further probe its potential clinical utility, CRC primary tumor cells and organoids from 12 patients were cultured and treated with APA (Fig. 4c). By genotyping *NAT2* in each sample, we attempted to establish an association between predicted NAT2 acetylator phenotype and response to drug treatment (Supplementary Table 6). In general, we found that tumors identified as homozygous for the slow acetylator variants NAT2*5 and *6 were more sensitive to APA treatment than tumors encoding rapid (NAT2*4 or *13) or intermediate (NAT2*4/*5B or NAT2*12C/*6F) acetylator phenotypes. To assess whether somatic or constitutional mutations in *AURKA* or other target kinases are likely to affect APA response, we turned to publicly available databases. In colonic and rectal adenocarcinomas cataloged in COSMIC, only 1.7% had somatic mutations in *DYRK1A*, 1% in *AURKA*, and none in *CDK7*. Further, the somatic mutations observed were scattered across the coding sequences indicative of background mutations. Next, we analyzed gene expression levels in the 66 CRC cell lines of the CCLE Public expression dataset 19Q3 [https://depmap.org/portal/] and observed that *AURKA*, *DYRK1A*, and *CDK7* had low-intermediate *AURKA* expression (Supplementary Fig. 8). We therefore conclude that APA response in our model systems are not dependent on high AURKA expression levels.

## Discussion

In this work, we identify and prioritize target genes for exploiting recurring LOH events in common tumor types. Loss of *NAT2*, a non-essential enzyme involved in drug metabolism, in colorectal cancers emerged as an attractive target conceptually different from synthetic lethality (*ENO2, PRMT5, ME3*) or targeting genes essential for cell survival (*POLR2A, RPA70*, or *PSMC2*). The findings here support that loss of rapid NAT2 in ~4% of chromosomally instable tumors from patients initially heterozygous for rapid and slow NAT2 is targetable by allele-specific inhibition using APA, a kinase inhibitor whose targets include Aurora A (Fig. 4d). While ~50,000 new CRC cases per year globally could potentially benefit from a NAT2 LOH-directed approach, this study also implies that drug metabolic enzymes are attractive targets for allele-specific inhibition strategies as their substrate specificity is well known allowing remarkably efficient selection of compounds for drug discovery. As a majority of slow NAT2 primary tumor cell cultures are inhibited by APA, these findings motivate studies determining whether the cohort of targetable patients should include tumors retaining slow acetylator *NAT2* variants other than *6 after undergoing LOH. Clinical sequencing can now inform the oncologist of the loss of specific drug metabolic activities in tumor cells relative to normal cells of the individual patient. Cytotoxic agents and kinase inhibitors tailored for inactivation by such activities can be expected to have wider therapeutic windows, in particular when combined with tumor targeted delivery vehicle such as liposomes, and allow for higher dosing and thereby higher initial cure rates.

## Methods

### Identification of nsSNVs in active surfaces of enzymes

Data for human genetic variation were obtained from Phase 1 samples of the 1000 Genomes project (Release 3, 2012-04-30). The consensus coding DNA sequence definitions of human protein coding exons were retrieved from the CCDS database (Release 15, 2013-11-29). The exon definitions were used to select all SNVs which reside in protein coding regions. Genetic variants with allele frequency <0.005 or >0.995 were discarded. To map the remaining SNVs to functional protein domains, we translated genome coordinates into protein coordinates and retained non-synonymous SNVs for which the protein coordinates mapped to protein sequences from the UniProtKB database (Release 2013_12). The resulting set of SNVs was enriched for potential relevance to catalytic activity by matching to the functional domain definitions in the Pfam 27.0 database (Release 2013-03-15). Next, all SNVs mapping to protein domains with an annotated active site were visually inspected if a crystal structure of the domain was available in the PDB database. Finally, SNVs generating substitutions of a surface exposed amino acid in close proximity to active site residues or substrate binding sites were selected for further studies. The tissue and tumor type specific gene expression profiles were obtained from IST Online database^20^ for exclusion of candidate genes, with expression below 300 in the tissues of origin of common cancers. The frequencies of LOH at specific loci in common human solid tumors were estimated from ref. ^15^. The estimate of eligible patients (EP) per target gene was based on the frequency of heterozygotes (HF) for each SNV in 1000 Genomes, the LOH frequency (LF) and the yearly worldwide incidence (I) of the respective cancer type (Cancer Research UK, 2012), and was calculated as EP = 0.5 × HF × LF × I assuming that only one of the two alleles is an exploitable therapeutic target. All relevant computer code is available upon request.

### Genotype of potential LOH therapy targets in human CRCs

The analysis of patient-matched tumor-normal pairs was approved by the Regional Ethical Review Board of Uppsala (2007/116, 2010/198). Tumor and patient-matched normal DNA from 74 chromosomally unstable CRCs (Supplementary Table 2) were genotyped by PCR coupled Sanger sequencing to determine the prevalence of loss of heterozygosity of six single nucleotide variants (SNVs) located in the candidate genes *NAT2* (rs1799930 and rs1801280), *ABP1* (rs1049793), *AKR7A2* (rs1043657), *SULT1C3* (rs2219078), and *SULT1A1* (rs9282861). Primers covering each variant region were designed using Primer3^34^ (Supplementary Table 7). Each PCR was performed in 20 μL reactions containing 1× Phusion HF Buffer (Thermo Scientific), 0.2 mM dNTPs, 0.2 μM forward, and 0.2 μM reverse primers, 0.02 U μL^−1^ Phusion DNA Polymerase (Thermo Scientific) and 6 ng of genomic DNA. The PCR was performed in a 2720 Thermal Cycler (Applied Biosystems) and consisted of 1 cycle of 98 °C for 30 s and 30 cycles of 98 °C for 10 s, 61 °C for 15 s and 72 °C for 20 s followed by 1 cycle of 72 °C for 10 min. For sequencing the PCR products, 18 μL reactions were prepared containing 20 ng of the template DNA and the corresponding forward or reverse amplification primer (4 pmol) for each SNV and sequenced on an ABI 3630xl instrument (Applied Biosystems). The sequences for tumor and patient-matched normal samples were analyzed using GeneScanner v1.0 (Applied Biosystems).

### Generation of CRC cells expressing rapid and slow NAT2

The human colorectal cancer cell lines RKO and DLD-1 (ATCC) were maintained in McCoy’s 5 A medium supplemented with 10% Fetal Bovine Serum (FBS) and 1% Pen Strep (Life Technologies). The coding sequence of *NAT2* was determined in the human CRC cell lines RKO and DLD-1 to determine if they encode a slow NAT2 variant. Genomic DNA from each cell line was isolated using a standard protocol for human or animal tissue or cultured cells (Macherey-Nagel) and amplification of the region of interest was performed by PCR using forward primer 5′-GGCTGTTCCCTTTGAGAACC-3′ and reverse primer 5′-AAATGCTGACATTTTTATGGATGA-3′ spanning exon 2 of *NAT2*. Lack of *NAT2* expression was confirmed in the chosen CRC cell lines by RT-qPCR, using forward primer 5′-GCAGACTTTGGAAGGGAGAG-3′ and reverse primer 5′-CAATGTCCATGATCCCTTTG-3′ for *NAT2* and forward primer 5′-CGGCTGTTTAACTTCGCTTC-3′ and reverse primer 5′-CACACGCCAAGAAACAGTGA-3′ for the reference gene *TBP*. Complementary DNA from the human liver cancer cell line HepG2 was used as positive control as it expresses *NAT2*^23^. Data were analyzed by the ΔΔCt method using the StepOne™ Software v2.1 (Thermo Fisher Scientific). The cell lines used in this paper are not listed in the database of commonly misidentified cell lines (ICLAC). The cell lines were authenticated by STR profiling (ATCC cell authentication service) and regularly checked for mycoplasma infection with MycoAlert mycoplasma detection kit (Lonza).

### Generation of expression vectors for rapid and slow NAT2

To generate slow and rapid NAT2 acetylator variants, the pCMV6-Entry-NAT2 mammalian expression vector containing a Myc-tagged *NAT2* open reading frame under a CMV promoter was acquired (OriGene). Sequencing of pCMV6-Entry-*NAT2* revealed a slow acetylator haplotype (rs1799930 G>A, NAT2*6A). To obtain a rapid acetylator phenotype (rs1799930 A>G, NAT2*13A), site-directed mutagenesis of rs1799930 was performed using the QuickChange II Site-Directed Mutagenesis Kit (Agilent Technologies) in 50 μL reactions containing 1 × reaction buffer, 125 ng of each primer, 0.2 mM dNTPs and 20 ng of dsDNA template. Primers were designed using the QuikChange algorithm (Agilent Technologies) and consisted of forward 5′-GAAGAGGTTGAAGAAGTGCTGAGAAATATATTTAAGATTTCCTTGG-3′ and reverse 5′-CCAAGGAAATCTTAAATATATTTCTCAGCACTTCTTCAACCTCTTC-3′. To generate a control plasmid, the *NAT2* ORF was excised from pCMV6-Entry-*NAT2* by BamHI digestion followed by vector re-ligation. For DNA digestion, 1 μg of dsDNA in 1 × FastDigest® buffer was incubated with 1 U BamHI FastDigest® enzyme (Thermo Scientific) at 37 °C for 90 min. The restriction enzyme was inactivated through incubation at 80 °C for 10 min. Ligation of the resulting linear DNA vector was performed with the T4 DNA Ligase enzyme (Thermo Scientific). Briefly, a 50 μL reaction was prepared by combining 10 μg of linear DNA, 1 × T4 DNA Ligase Buffer and 0.2 U μL^−1^ of T4 DNA Ligase. The sample was incubated at 22 °C for 10 min. The sequences of the three constructs were verified by Sanger sequencing.

The human CRC cell lines RKO and DLD-1 were maintained in McCoy’s 5 A medium supplemented with 10% Fetal Bovine Serum (FBS) and 1% Pen Strep (Life Technologies). Cells were grown to 80% confluence and transfected with each NAT2 acetylator variant expression vector using Lipofectamine® 2000 DNA Transfection Reagent (Invitrogen). After 48 h, RKO and DLD-1 clones were expanded under G418 selection (1 mg mL^−1^). The resulting clones were confirmed by PCR coupled Sanger sequencing using primers forward 5′-GATCCGGTACCGAGGAGAT-3′ and reverse 5′-TTGCTGCCAGATCCTCTTCT-3′, spanning a region of the expression vector containing the coding exon 2 of *NAT2* and designed to exclude the amplification of endogenous *NAT2* alleles. Next, PCR positive RKO and DLD-1 cell clones were confirmed by western blot. Clones were lysed with RIPA lysis buffer (25 mM Tris-HCl at pH 7.6, 150 mM NaCl, 1% NP-40 and 0.1% SDS) and centrifuged at 15,000 × *g* at 4 °C for 15 min. The supernatant was retained and protein concentration was determined by the BCA assay (Novex®, Life Technologies). Forty micrograms of each sample was loaded along with PageRuler™ Prestained protein ladder (Thermo Scientific) on a NuPAGE® 4–12% Bis-Tris gradient gel with 1 × NuPAGE® MES SDS running buffer (Novex®, Life Technologies) and separated at 180 V for 1 h. Transfer was done at 30 V for 2 h to a Hybond C-Extra membrane (Amersham Biosciences) in transfer buffer (48 mM Tris base, 39 mM glycine). The membrane was blocked in 5% non-fat dry milk in PBS for 1 h and immunoblotted overnight with primary antibodies rabbit α-Myc (71D10, Cell Signaling Technology), or mouse α-Tubulin (T8203, Sigma-Aldrich) diluted 1:1000 in PBS containing 3% BSA. The membrane was washed in PBS and further incubated for 1 h with goat anti-rabbit and goat anti-mouse secondary antibodies (31460 and 31430, Thermo Scientific) diluted 1:10,000 in PBS containing 3% BSA. Signal detection was performed with the SuperSignal West Femto kit (Thermo Scientific) in the ImageQuant LAS 4000 imager (GE Healthcare Life Sciences). Unprocessed immunoblot scans are provided in Supplementary Fig. 9.

### Quantification of NAT2 catalytic activity in cell lysates

RKO and DLD-1 cells were plated at a density of 18,000 cells per well and treated with 10 µM of the NAT2-specific substrates amonafide (Adooq Bioscience) and procainamide (Sigma-Aldrich). NAT2 catalytic activity was quenched by addition of 99.8% methanol to each well after 30 min and compound separation was achieved by liquid chromatography (LC) using a C18 BEH column (1.7 µm, 2 × 50 mm; Waters) with mobile phases consisting of 10 mM ammonium carbonate, pH 10 (A) and acetonitrile (B). NAT2 substrate and product levels were detected through tandem mass spectrometry (MS/MS) in a XEVO TQ mass spectrometer (Waters), by monitoring the following *m*/*z* transitions: 284>239 (amonafide), 326>281 (N-acetyl-amonafide), 236>163 (Procainamide), and 278>205 (N-acetyl-procainamide).

### Cell-based screen for cytotoxic drugs metabolized by NAT2

To enrich for potential NAT2 substrates, the database of the compound collection (189,018 compounds) of CBCS (Chemical Biology Consortium Sweden) was analyzed for primary arylamines yielding 1150 compounds of which 156 were available for screening. Of the 156 compounds, 120 had shown cytotoxicity in previous cell-based screens at CBCS. In addition, 20 primary heteroarylamines that had previously shown cytotoxicity were included. In total, 176 compounds were selected based on chemical structure and their potential to impair cell growth. Each of the compounds was evaluated at three concentrations (0.5, 2, and 10 μM) using a rapid acetylator RKO clone and a vector control clone. Cells were seeded at 8000 cells per well in 96-well plates and incubated with the different compounds for 72 h. Next, cell viability was scored in an MTT assay according to the manufacturer’s protocol by addition of resazurin (Sigma-Aldrich) to each well and incubation of the plates at 37 °C. Fluorescence was read at 590 nm 2 h following the addition of resazurin using a Victor^2^ 1420 multilabel counter (Wallac).

### Quantification of NAT2 catalytic activity toward APA

RKO and DLD-1 cells were plated at a density of 18,000 cells per well in a 96-well plate and treated with 0.1–50 µM of APA (AKos GmbH). The NAT2 catalytic activity was quenched after 30 and 60 min (0.1–1 µM APA) or after 15 and 30 min (2–50 µM APA) by addition of 99.8% methanol. APA and N-acetyl APA (NAPA, AKos GmbH) were detected by LC-MS/MS on a XEVO TQ (Waters) coupled to an Acquity UPLC (Waters) using a HSS T3 column (1.7 µm, 2 × 50 mm; Waters). The mobile phases consisted of 0.05% heptafluorobutyric acid and propionic acid (A), and 0.05% heptafluorobutyric acid and propionic acid in acetonitrile (B). The following *m*/*z* transitions were monitored: 353>323 (APA) and 395>143 (NAPA).

### In vitro ADME profiling of APA and NAPA

Metabolic stability was assessed by incubating APA and NAPA at a final concentration of 1 μM with 0.5 mg⋅ml^−1^ pooled CD-1 mouse liver microsomes (XenoTech, cat. no. M1000) in 100 mM potassium phosphate buffer pH 7.4. The reaction was initiated by addition of NADPH at a final concentration of 1 mM and stopped at 0, 5, 10, 20, 40, and 60 min by transferring a 50 µl sample to 100 μl ice-cold acetonitrile. Before LC-MS/MS analysis, samples were centrifuged at 3000 × *g* for 20 min at 4 °C. In vitro half-life was estimated as previously described^35^. Bufuralol, diclofenac and midazolam were used as controls. Solubility in phosphate buffer was determined by adding 5 μl of 10 mM DMSO stock solution of the compounds to 500 μl of 100 mM potassium phosphate buffer pH 7.4. After overnight incubation of the samples at 37 °C, the samples were centrifuged at 3000 × *g* for 30 min at 37 °C and analyzed by LC-MS/MS. Ketoconazole and nicardipine were used as controls. Plasma protein binding was measured by incubating the compounds at a final concentration of 10 μM with pooled CD-1 mouse plasma (Innovative Research) in a Rapid Equilibrium Dialysis device (Thermo Fisher Scientific) against isotonic 67 mM phosphate buffer pH 7.4. After 4 h incubation at 37 °C, samples were 10-fold diluted in acetonitrile. Before LC-MS/MS analysis, samples were centrifuged at 3000 × *g* for 20 min at 4 °C. Fraction unbound in plasma was calculated as the ratio of compound concentration in the buffer sample and the plasma sample. Diclofenac and propranolol were used as controls.

### Binding affinities of APA and NAPA toward protein kinases

The binding affinities of APA and NAPA were evaluated in the *scan*MAX^sm^ Kinase Assay Panel (DiscoverX) at a final concentration of 10 µM. The compounds were tested for steric or allosteric binding to kinases in solution. The kinase was captured from the solution through an immobilized kinase-specific ligand and the kinase recovery was quantified. The amount of recovered kinase in the presence or absence of APA or NAPA was measured by quantitative PCR compared with the kinase capture in absence of the compound. Any compound reducing the binding affinity of a kinase to the respective immobilized ligand was scored as a potential hit, and the binding affinity of the compound toward a kinase is interpreted as the difference between the kinase recovery in the absence and presence of the compound. The inhibitory binding constants (*K*_d_) of APA and NAPA to AURKA were evaluated in a *Kd*ELECT Kinase Assay (DiscoverX). The *K*_d_ was derived from independent duplicate of 11-point dose-response curves ranging from 0.85 nM to 50 µM for each compound. Data acquisition followed the same methodology as for the *scan*MAX^sm^ Kinase Assay Panel.

### siRNA knockdown of *DYRK1A*, *AURKA*, and *CDK7*

The RKO and DLD-1 CRC cells were plated at 10,000 or 5000 cells, respectively, per well in a 96-well plate. After 16 h, the cells were exposed to serum- and antibiotics-free McCoy’s 5A medium and transfected with SMARTpool ON-TARGETplus control siRNA or siRNAs targeting *DYRK1A*, *AURKA*, or *CDK7* using the DharmaFECT 2 transfection reagent (Dharmacon). After 5 h the cells were supplemented with FBS to 10% final concentration and incubated for 72 h. The cell viability was evaluated in an MTT assay as described above.

### siRNA knockdown of *NAT2* in HCT116 cells

HCT116 (ATCC) CRC cells were plated at 4500 cells per well in a 96-well plate, let attach for 16 h and transfected with SMARTpool ON-TARGETplus control siRNA or siRNAs targeting *NAT2* using the DharmaFECT 2 transfection reagent (Dharmacon) in serum- and antibiotics-free McCoy’s 5A medium. After 5 h, the cells were supplemented with FBS to 10% final concentration and incubated for 24 h. Finally, cells were treated with APA or 5-FU in different concentrations and cell viability was evaluated in an MTT assay after 68 h.

### RT-qPCR analysis

Direct cDNA extractions were performed from cell lysates using the TaqMan® Gene Expression Cells-to-CT™ Kit (Thermo Fisher Scientific). The expression of *NAT2*, *DYRK1A*, *AURKA*, or *CDK7* transcripts was detected by qPCR using TaqMan probes against *NAT2* (Hs04194721_s1), *DYRK1A* (Hs00176369_m1), *AURKA* (Hs01582072_m1), or *CDK7* (Hs00361486_m1) with *ACTB* (Hs01060665_g1) as an internal reference gene. The quantification was performed according to the comparative Ct (ΔΔCT) standard protocol in StepOnePlus instrument (Applied Biosciences).

### Validation of APA as a NAT2-specific substrate

The acetylation rate of APA (AKos GmbH), 4-aminoasylic acid (PAS) (Sigma-Aldrich) and procainamide (Sigma-Aldrich) was determined by incubation with recombinant human N-acetyltransferases 1 and 2 (Corning-Supersomes™). Briefly, triethanolamine pH 7.5 (50 mM), acetyl-CoA (0.1 mM), acetyl-d,l-carnitine (4.6 mM), EDTA (1 mM), DTT (1 mM), and carnitine acetyltransferase (0.6 U/mL) were combined with 10 µM of the substrate. After incubation at 37 °C for 5 min, the enzymatic reaction was initiated by addition of 0.005 mg/ml of the respective recombinant NAT enzyme. NAT activity was quenched by addition of 200 µl of acetonitrile to 100 µl of the incubation mix at indicated timepoints followed by centrifugation at 10,000 × *g* for 3 min and analysis of the supernatant. NAT2 catalytic activity toward APA and NAPA was quantified by LC-MS/MS as described above.

### Liposomal preparation of APA

A mixture of HEPC:Chol:DSPE-PEG_2000_ at a molar ratio of 50:45:5 was solubilized in chloroform, dried overnight to a thin film under rotary evaporation, hydrated with 1 M sodium citrate (pH 3) and submerged into a 65 °C sonication bath to allow formation of large multilamellar vesicles. The lipid suspension was extruded 10 times through a double stack of 0.1 μm Nuclepore filters (Whatman) using a Lipex Thermobarrel Extruder (Northern Lipids). The resulting colloidal suspension of single unilamellar vesicles (SUVs) was then dialyzed against 300 mM sucrose at 4 °C. The mean size of the SUVs was determined by quasi-elastic light scattering using a Malvern Zetasizer 3000 (Malvern). APA was actively loaded into liposomes using a citrate pH gradient. To encapsulate APA, the compound was dissolved in DMSO and mixed with liposomes at a drug:lipid molar ratio of 1:3 following incubation at 65 °C for 30 min. Drug-loaded liposomes were stored at 4 °C and diluted to 8.8 mg mL^−1^ of APA in PBS before use. Encapsulation efficiency was 95.5% as determined by disruption of liposomes with 0.1% Triton X-100 and fluorometric measurement (excitation at 380 nm, emission at 450 nm) using a fluorescence plate reader (Tecan Infinite 200). Concentrations were derived by reference to an APA standard curve. Free APA was prepared by solubilization with HCl followed by dilution with PBS to achieve a final concentration of 8.8 mg mL^−1^.

### APA treatment of mouse xenograft models

The BALB/c nude mice are rapid Nat acetylators (Nat1*6, Nat2*8, and Nat3*1), and the scarcity of genetic polymorphisms in the *Nat* mouse genes^36^ supports the biological relevance of this animal model in recreating APA targeting in a NAT2-proficient organism. Mouse *Nat2* and human *NAT1* are orthologous and believed to share similar substrate specificity and tissue expression profiles^37^. All animal experiments were overseen and approved by the Institutional Animal Care and Use Committee of Temasek Life Sciences Laboratory at the National University of Singapore (#TLL-14-022). Statistical power analysis was performed to determine the minimum number of animals per experimental arm required to demonstrate a statistically significant difference in treatment outcomes. Dose escalation studies revealed 88 mg kg^−1^ as maximum tolerated dose of free APA. Six to eight week female NCr nude mice (In Vivos) were each injected subcutaneously with 4 × 10^6^ RKO NAT2 slow cells in the left flank and 4 × 10^6^ RKO NAT2 rapid cells on the right flank. The xenografts were grown for 10 days before randomization and non-blinded treatment. A minimum of eight animals were used for each experimental arm, consisting of liposomal APA (88 mg kg^−1^), free APA (88 mg kg^−1^) and control treatment (empty liposomes suspended in PBS). Tumor volumes were calculated as length × width^2^ × 0.5. All drug and control treatments were administered at the indicated timepoints as tail vein injections in volumes of 0.2 mL. Weight loss remained <10% during the lapse of the experiment and did not differ significantly between treatment types (Supplementary Fig. 7D).

### Treatment of primary cells and organoids from CRC patients

Tumor samples were obtained from CRC patients operated for peritoneal metastasis. The patient sampling was approved by the regional ethical committee, Uppsala University (file Dnr 2007/237) following participant consent. Tumor cells were prepared by finely mincing of the tissue followed by collagenase digestion and Percoll (GE Healthcare) density gradient centrifugation^38^. Organoid samples from CRC patients were obtained after participant consent and approval from the Gastrointestinal Cancer Center at the Dana-Farber Cancer Institute (protocol numbers 03-189, 17-000, and 18-060). Samples were grown in Advanced DMEM/F12 medium (Thermo Fisher), supplemented with 4 mM HEPES (Life Technologies), 1 mM GlutaMax (Life Technologies), 50 U mL^−1^ PenStrep (Thermo Fisher), 1× B27 (Thermo Fisher), 100 ng mL^−1^ Noggin (BioLegend), 50 ng mL^−1^ EGF (Life Technologies), 1 mM N-acetylcycteine (Sigma-Aldrich), 500 nM A-83-01 (Fisher Scientific), 10 µM SB202190 (Sigma-Aldrich), 10 mM nicotinamide (Sigma-Aldrich), 10 nM [Leu15]-Gastrin I (Sigma-Aldrich), and 100 µg mL^−1^ Primocin (InvivoGen). After specimen collection, human tumor tissue samples were placed on a 10 cm dish on ice and minced with a No. 10 scalpel prior to incubation for 1 h at 37 °C in CCOM medium consisting of 5 mg mL^−1^ collagenase XI (Sigma-Aldrich), 10 µg mL^−1^ DNase I (StemCell) and 10 µM Y-27632 (Sigma-Aldrich). The cell suspension was then centrifuged at 500 × *g* × 5 min and washed twice with CCOM medium. The pellet was seeded at a density of 2000 cells per well, each containing 50 µL Matrigel (Corning). Media was replaced every 2–3 days and organoids were passaged following TrypLE Express (Thermo Fisher) incubation for 15–20 min at 37 °C.

For assessment of drug activity, 45 µl cell suspension (5000 cells per well) was seeded into each well in the drug-prepared 384-well plates (5 µl per well of drug solution at 10× final concentration). The culture plates were incubated for 72 h at 37 °C in 5% CO_2_ before assessment of cell viability using the fluorometric microculture cytotoxicity assay (FMCA) based on the conversion of fluorescein diacetate (Sigma-Aldrich) to fluorescein by esterases in cells with intact plasma membranes^38^. For organoid cultures, cells were dissociated using TrypLE Express (Thermo Fisher), centrifuged at 500 × *g* for 5 min and washed with CCOM medium. Next, cells were seeded in a 96-well plate in DMEM medium (Thermo Fisher) containing 10% Matrigel (Corning) at a cell density of 10,000 cells per well. After 48 h, CCOM medium containing APA was added to the organoids. ATP activity was measured as a surrogate marker of cell viability after 72 h using the CellTiter-Glo 2.0 kit (Promega) according to the manufacturer’s instructions.

### Thermal shift assay with human recombinant Aurora kinase A

Recombinant human Aurora Kinase A (AURKA) (SRP0354, Sigma) was dissolved in PBS at a concentration of 0.1 mg mL^−1^ and mixed with the positive control AURKA Inhibitor I (S1451, Selleckchem) or APA at a final concentration of 10 µM. Thermal unfolding experiments were performed in a Prometheus NT.48 instrument (NanoTemper) by loading 10 µL of sample into nanoDSF Grade High Sensitivity Capillaries (PR-C006, NanoTemper Technologies) and setting a temperature gradient of 1 °C increase per min in a range from 20 to 90 °C. Protein unfolding was detected by the change in tryptophan fluorescence at 330 nm and 350 nm, and melting temperatures (Tm) were calculated by detecting the first derivative of the fluorescence ratio at both wavelengths (F350/F330).

### Immunofluorescence stainings

Cells were fixed in ice-cold methanol for 4 min and washed with TBS supplemented with 0.1% Tween. Blocking was performed with normal donkey serum (Jackson Immuno Research) for 1 h at room temperature and cells were incubated overnight with mouse anti-α-tubulin (1:100, T9026, Sigma-Aldrich), rabbit anti-phospho-Aurora A (1:100, C39D8, Cell Signaling Technology) and rabbit anti-phospho-histone H3 (pSer10) (1:100, 04-817, Merck) monoclonal primary antibodies at 4 °C. Next, cells were incubated with the fluorescently labeled secondary antibodies Alexa-488 donkey anti-mouse (1:500, A21202, Invitrogen) and Alexa-467 donkey anti-rabbit (1:500, A31573, Invitrogen) for 1 h at room temperature. Slides were washed and nuclei were stained with Hoechst 33342 (1:5000, H3570, Molecular Probes) before mounting with Fluoromount-G mounting medium (SouthernBiotech). Imaging was done in a Leica SP8 confocal microscope using the Leica application suite X fluorescence software. Image acquisition was done with a ×63 objective, followed by processing and quantification with ImageJ (NIH) and CellProfiler (Broad Institute).

### Immunoblotting for AURKA detection

The standard protocol (described above) for cell lysis and western blot was followed with exception that the membrane washes were in tris-buffered saline with 0.1% Tween 20 (TBS-T) and the membrane blocking and antibody probing was performed with 5% BSA in TBS-T. The primary antibodies used for protein detection were rabbit monoclonal anti-Aurora A (1:10,000, ab52973, Abcam), mouse monoclonal anti-PCNA (1:4,000, PC10, sc-56, Santa Cruz Biotechnology), rabbit monoclonal anti-phospho-histone H3 (pSer10) (1:10,000, 04-817, Merck) and rabbit polyclonal anti-histone H3 (1:10,000, ab1791, Abcam). The secondary antibodies were goat anti-rabbit and goat anti-mouse (31460 and 31430, Thermo Scientific) diluted 1:10,000 in TBS-T containing 5% BSA. The immunoblot quantification was performed by ImageJ software (NIH) and the data for normalization to the loading control were obtained following the integration of the band intensity. Unprocessed immunoblot scans for AURKA expression are provided in Supplementary Fig. 9.

### Reporting summary

Further information on research design is available in the Nature Research Reporting Summary linked to this article.

## Supplementary information

Supplementary Information

Description of Additional Supplementary Information

Supplementary Data 1

Reporting Summary

## Data Availability

The authors declare that the data supporting the findings of this study are available within the paper and its supplementary information files. The germline variation data and definitions for the filtering criteria used for the generation of Fig. 1a are available in public repositories at the following websites (ftp://ftp-trace.ncbi.nih.gov/1000genomes/ftp/phase1/analysis_results/integrated_call_sets/, ftp://ftp.ncbi.nlm.nih.gov/pub/CCDS/archive/15/, https://www.uniprot.org/news/2013/12/11/release, ftp://ftp.ebi.ac.uk/pub/databases/Pfam/releases/Pfam27.0/). Raw data underlying Figs. 2 and 3 as well as Supplementary Figs. 2, 4, and 5 are provided as a Source Data file. All other data supporting the findings of this study are available within the article and its supplementary information files and from the corresponding author upon reasonable request. A reporting summary for this article is available as a Supplementary Information file.

## Source Data

Source Data
