# Supplementary material for: Exploiting loss of heterozygosity for allele-selective colorectal cancer chemotherapy
Source: Nat Commun. 2020 Mar 11;11:1308. doi: 10.1038/s41467-020-15111-4 (PMC7066191; doi:10.1038/s41467-020-15111-4)
Supplement: Supplementary file 1 — Supplementary Information [file 41467_2020_15111_MOESM1_ESM.pdf]

Supplementary Information

Exploiting loss of heterozygosity for allele-selective colorectal cancer chemotherapy

Rendo and Stoimenov et al.

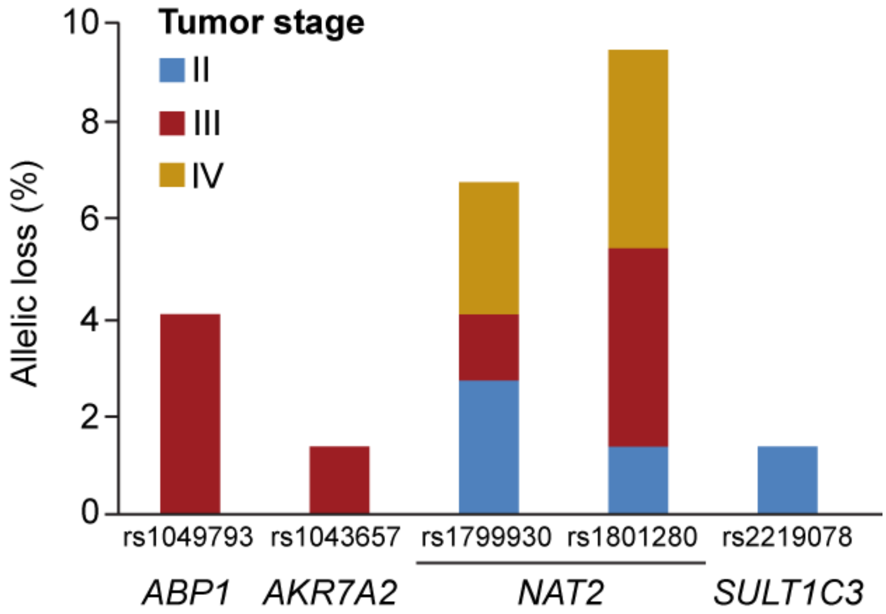

Supplementary Figure 1. LOH events in NAT2 are present in early stages of colorectal tumor progression

Each bar represents the percentage of allelic loss reported for a particular SNV in the genotyped CRC cohort, where a total of 17 LOH events were identified (*ABP1* = 3; *AKR7A2* = 1; *NAT2* = 12; *SULT1C3* = 1). Within each bar, the fraction of samples belonging to each stage of tumor progression is shown.

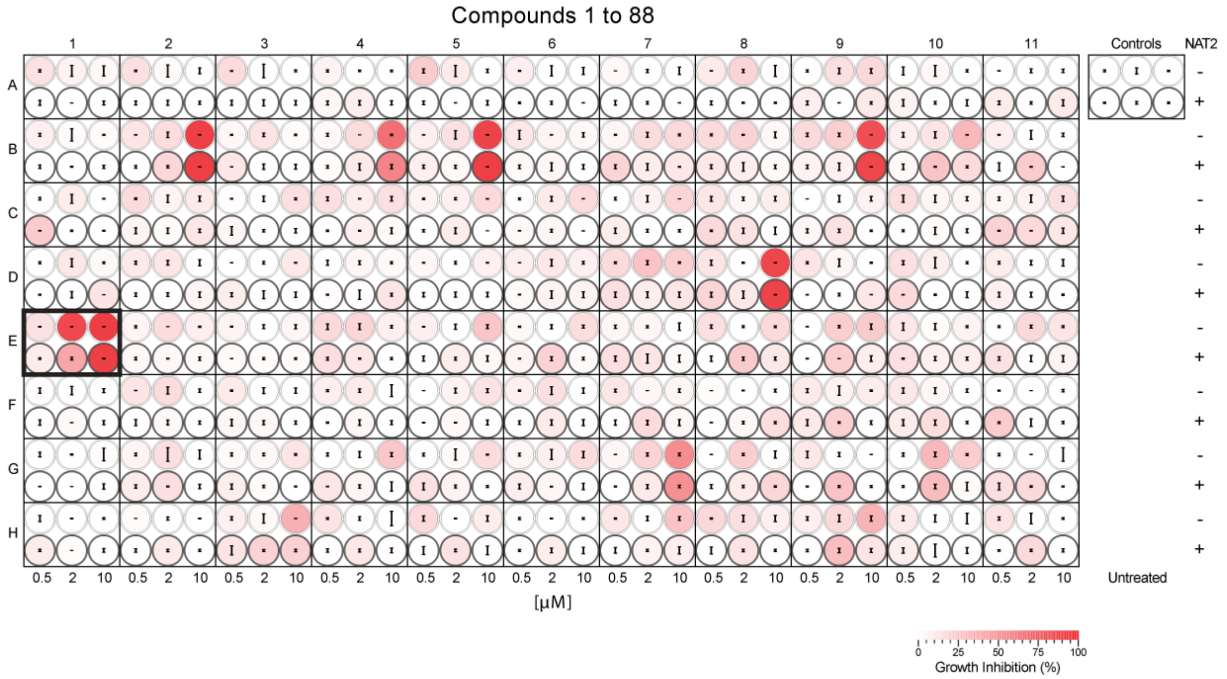

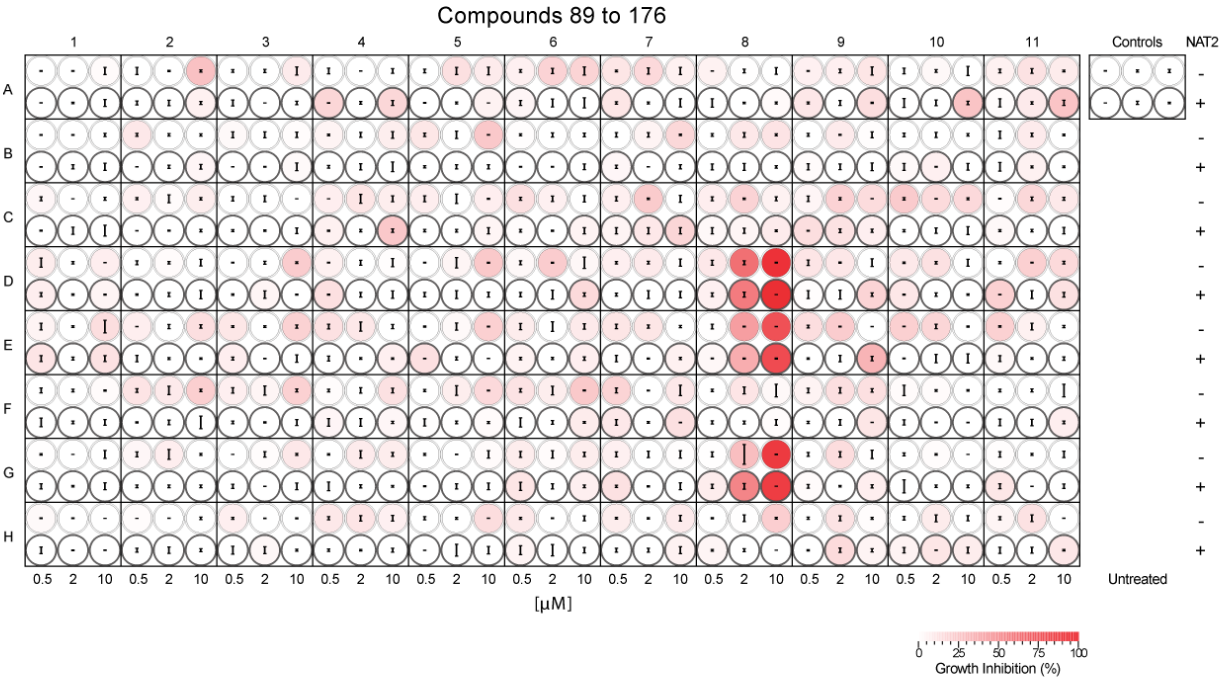

Supplementary Figure 2. A cell-based screen to identify cytotoxic compounds metabolized by NAT2

A set of 176 compounds were identified as potential NAT2 substrates by informatic analyses of a library of 189,018 chemical structures. These compounds were incubated at three concentrations (0.5 μM, 2 μM and 10 μM) with vector control (-) or rapid NAT2 expressing (+) RKO cells for 3 days and cell viability was scored in an MTT assay. The data for each compound are grouped with color intensity representing percentage growth inhibition relative to untreated controls (right panel, A). Black rectangle, hit compound (APA). Error bars in the center of the well, S.D. of two biological replicates.

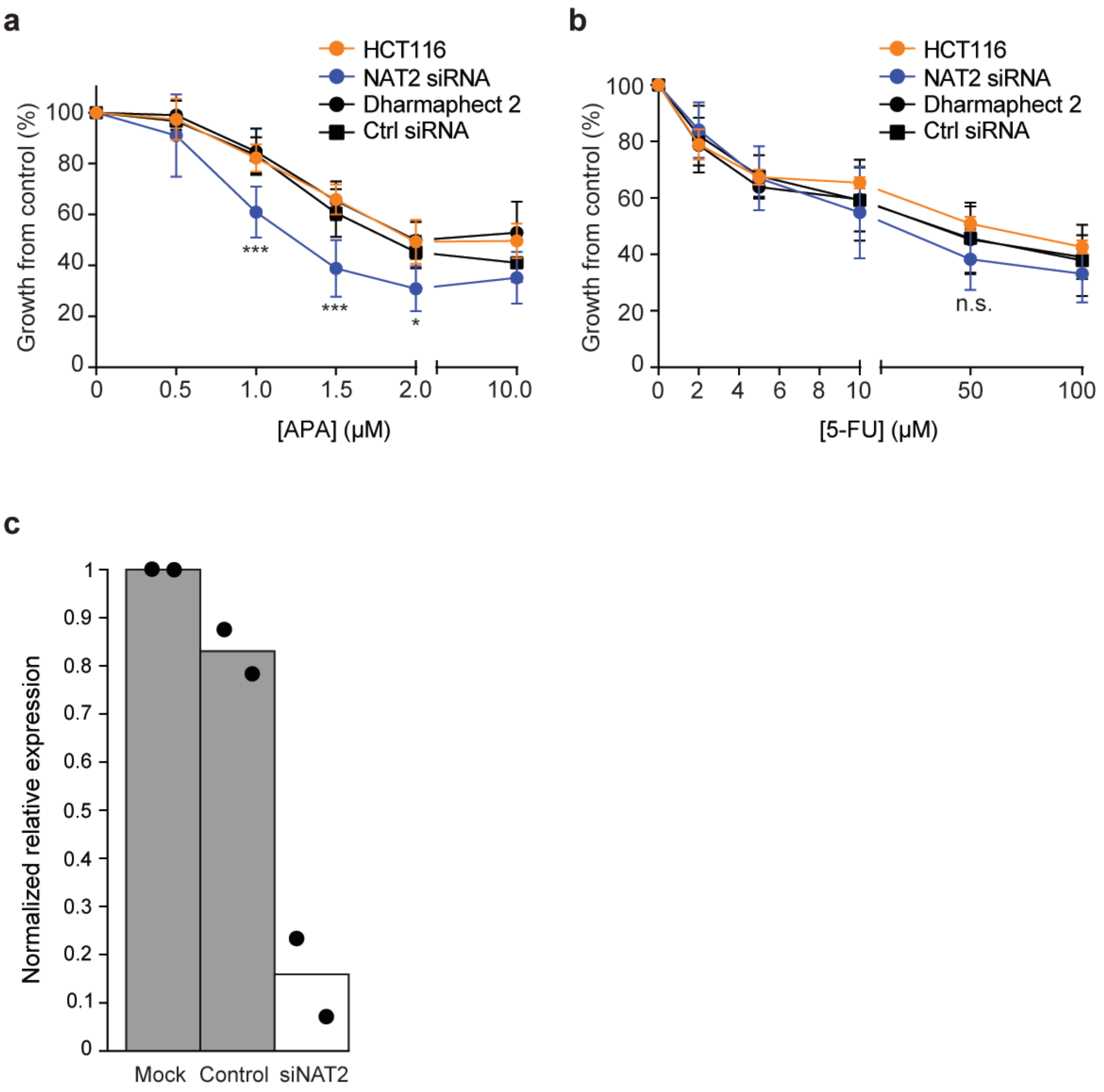

Supplementary Figure 3. Silencing of endogenous *NAT2* sensitizes HCT116 cells to APA treatment

HCT116 cells were transfected with a pool of siRNA targeting *NAT2*. Non-target RNA control, mock transfection and non-transfected cell control were included in the experiment. Following siRNA-mediated silencing of *NAT2*, cells were treated with **a** APA and **b** 5-FU. Mean and S.D. of 3 independent experiments. Data were analyzed using two-way ANOVA. n.s., $P = 0.4978$, *, $P = 0.0355$ and ***, $P = 0.006$ (1.0 μM) and 0.0008 (1.5 μM). **c** HCT116 cells show reduced transcript levels of *NAT2* following siRNA-mediated targeting. Cells were mock-treated, non-target RNA treated and treated with target siRNA against NAT2. A result of qPCR analysis of two independent experiments is shown, with 3 technical repeats in each. Means of normalized expression levels toward mock-treatment.

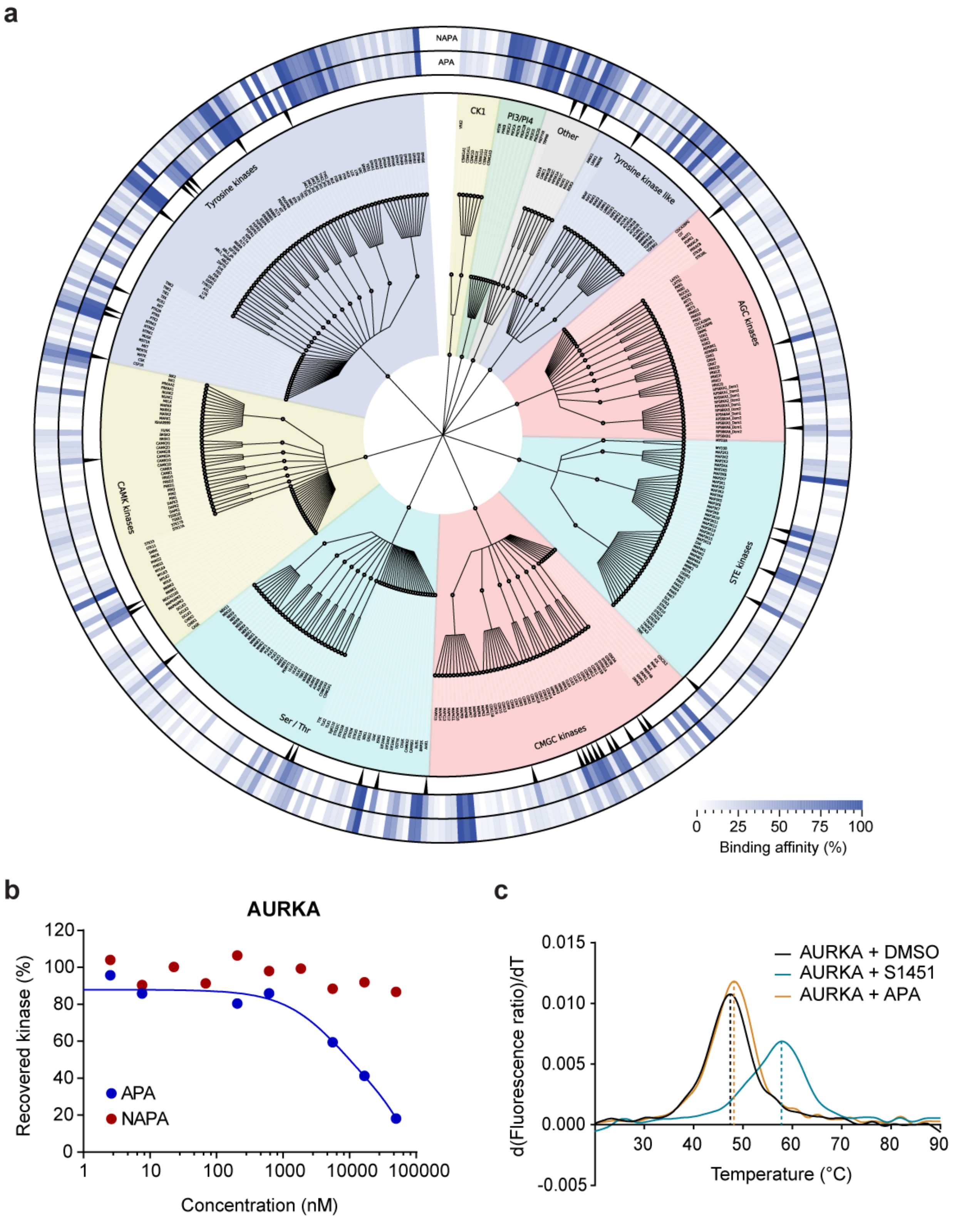

a
NAPA
APA
CK1
PI3/PI4
Other
Tyrosine kinase like
AGC kinases
STE kinases
CMGC kinases
Ser / Thr
CAMK kinases
Tyrosine kinases
0
25
50
75
100
Binding affinity (%)
b
AURKA
Recovered kinase (%)
0
20
40
60
80
100
120
1
10
100
1000
10000
100000
Concentration (nM)
APA
NAPA
c
d(Fluorescence ratio)/dT
0.015
0.010
0.005
0.000
-0.005
30
40
50
60
70
80
90
Temperature (°C)
AURKA + DMSO
AURKA + S1451
AURKA + APA

Supplementary Figure 4. Kinome inhibition profiles of APA and its NAT2 metabolite NAPA identifies preferential APA kinase targets and differential binding to AURKA

a The human kinome was assessed for binding affinity to APA and NAPA at 10 µM concentration (468 kinases in the scanMAX Kinase Assay Panel, DiscoverX). The family stratification of kinases is shown as a cladogram. Circles visualize the binding affinity of NAPA and APA towards each kinase with a higher intensity of blue corresponding to a higher binding affinity. Arrowheads point to kinases, for which the binding affinity of NAPA is ≤25% than the binding affinity of APA. **b** The amount of recovered AURKA in KdELECT Kinase Assay in presence of APA or NAPA by compound concentration. The inhibitory binding constant for APA was estimated from two independent experiments, whereas NAPA did not show substantial change in binding affinity in the tested concentration range. One single data point from one experiment at a concentration 18.5 µM of APA was excluded as an outlier. **c** Thermal shift assay for human recombinant AURKA in the presence of APA and the selective inhibitor S1451. Melting temperatures were obtained by calculating the first derivative of the tryptophan fluorescence ratio at 350 nm and 330 nm and are shown for AURKA in the presence of S1451 (blue) and APA (yellow).

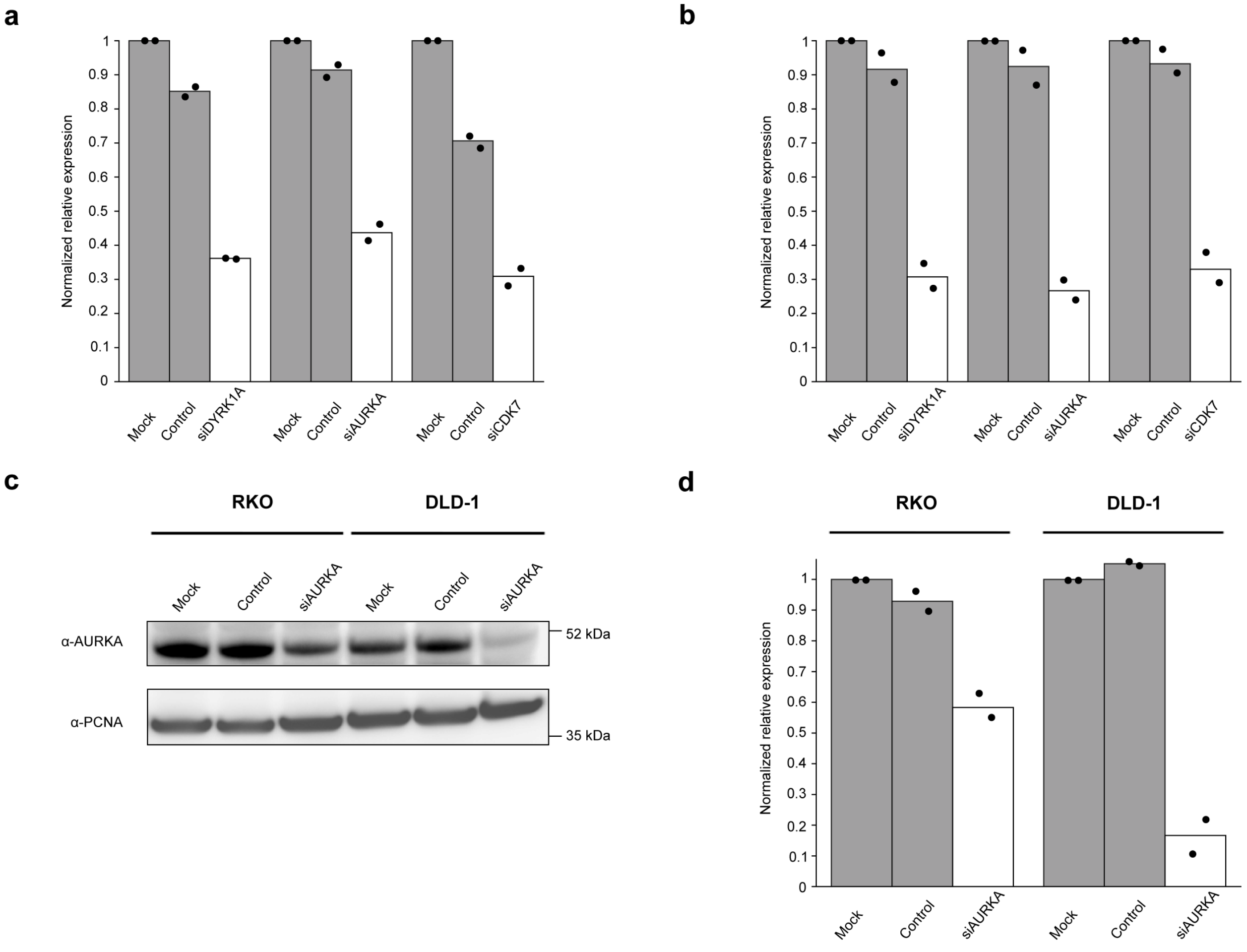

Supplementary Figure 5. siRNA-mediated knock-down reduces transcript levels of *DYRK1A*, *AURKA* or *CDK7* and AURKA protein levels in RKO and DLD-1 cells

RKO **a** and DLD-1 **b** cells show reduced transcript levels of *DYRK1A*, *AURKA* and *CDK7* following treatment with siRNA targeting the respective kinase. Cells were mock-treated, non-target RNA treated and treated with target siRNA against *DYRK1A*, *AURKA* or *CDK7*. A result of qPCR analysis of two independent experiments is shown, with 3 technical repeats in each. Means of normalized expression levels toward mock-treatment. **c** siRNA-mediated knockdown of *AURKA*, reduces the protein levels of AURKA in both RKO and DLD-1 cells. RKO and DLD-1 cells were subjected to immunoblot analysis with different antibodies against total AURKA and PCNA (loading control) following mock-treatment, non-target RNA control and siRNA targeting *AURKA*. **d** Protein expression levels of AURKA are reduced following siRNA-mediated silencing of *AURKA* in both RKO and DLD-1. Quantification of two immunoblot analyses performed as in **c**; AURKA protein levels were normalized to the loading control (PCNA) and relative expression towards mock treatment is shown as mean value.

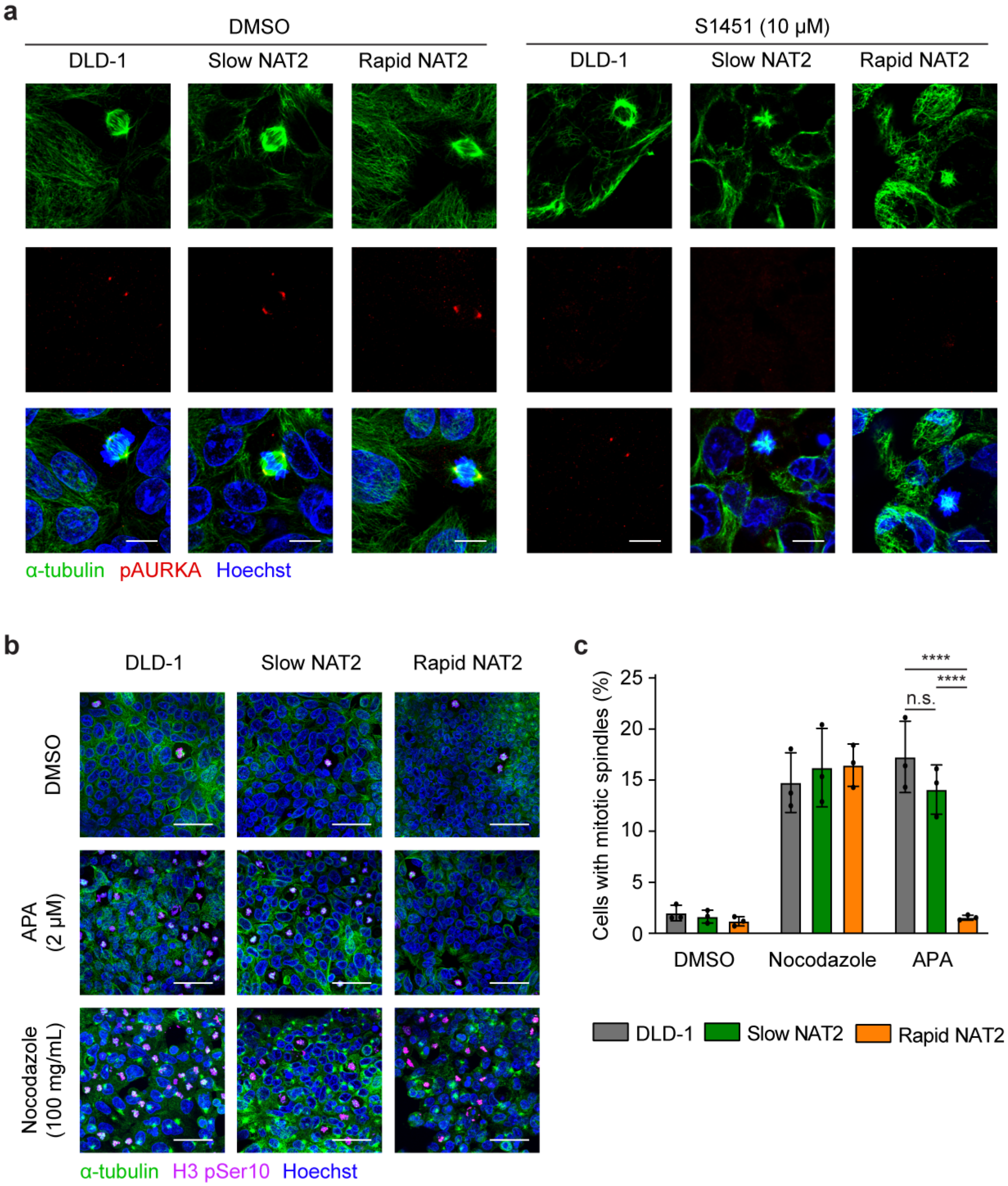

Supplementary Figure 6. APA reduces AURKA phosphorylation and causes mitotic arrest in NAT2-deficient cells

a Phospho-Aurora A levels in DMSO and S1451-treated DLD-1 cells. The pAURKA level (red) was detected in mitotic DLD-1 cells but not in parental DLD-1 cells and NAT2 clones treated with

the selective Aurora A kinase inhibitor S1451 (10 µM). Mitotic spindles were visualized by α-tubulin staining (green). Representative images of three independent repeats are shown for each cell type. Scale bar, 10 µm. **b** APA causes mitotic arrest in NAT2-deficient cells. Mitotic spindles were visualized by α-tubulin staining (green) and mitotic arrest was shown in histone H3 pSer10 positive cells (pink). The effect of APA was compared to nocodazole (100 ng/mL), a microtubule inhibitor that also causes mitotic arrest. Representative images of three independent repeats are shown for each cell type. Scale bar, 50 µm. **c** Quantification of cells with mitotic spindles after treatment with DMSO, APA (2 µM) or nocodazole (100 ng/mL). Mean and S.D. (error bars) of 3 independent experiments. Data were analyzed using two-way ANOVA. n.s., $P = 0.1857$ and ****, $P < 0.0001$.

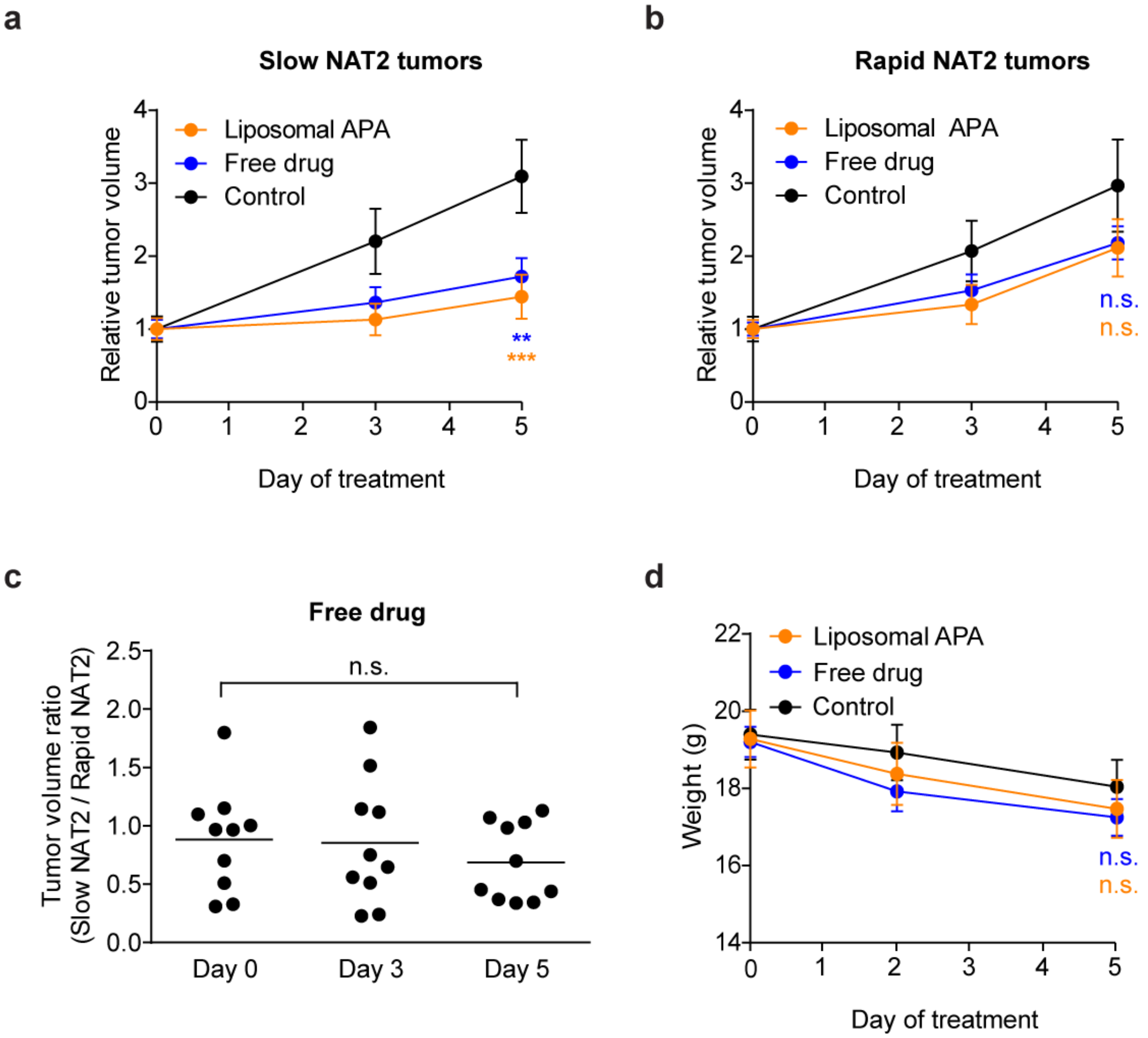

Supplementary Figure 7. APA treatment impairs the growth of slow NAT2 tumors

a, b The relative tumor volume of slow, but not rapid NAT2 tumors is decreased after 5 days of treatment with either liposomal or free APA. Tumor size was measured using a caliper and normalized to day 0 before treatment start. Data were analyzed using two-way ANOVA, n.s., P = 0.7734 (Liposomal APA) and 0.7157 (Free drug), **, P = 0.0028, ***, P = 0.0009. Mean and s.e.m. (error bars) shown for one representative experiment conducted with 10 mice for each treatment group. **c**. Treatment with APA as a free drug has no effect on tumor volume ratio. The rate at which slow NAT2 tumors grow compared to rapid NAT2 tumors is not significant in the group of animals treated with APA after 5 days. Data were analyzed using two-way ANOVA, n.s., P = 0.5708. Data points from one representative experiment conducted in 10 mice. **d** Animal weight remained stable during APA treatment. The weight of each animal was monitored during the course of the experiment. Data were analyzed using two-way ANOVA, n.s., P = 0.7918 (Liposomal APA) and 0.5755 (Free drug). Mean and s.e.m. (error bars) from one representative experiment conducted in 10 mice for each treatment group.

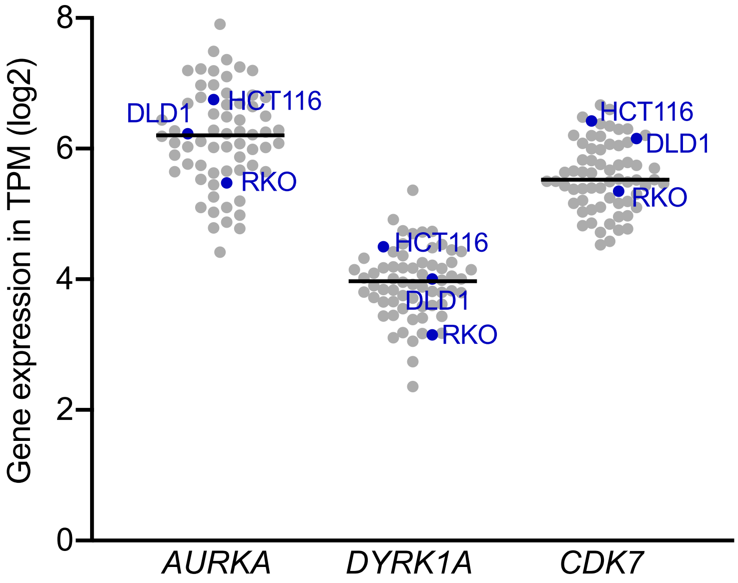

Supplementary Figure 8. Expression of AURKA, DYRK1A and CDK7 kinases in the CCLE colorectal cancer cell lines

The mRNA expression levels of the three kinases were obtained from the CCLE RNA-seq dataset (release 19Q3). Expression levels are represented as $\log_2$ TPM score. The cell lines used in this study are labeled in blue. Black lines, median gene expression values.

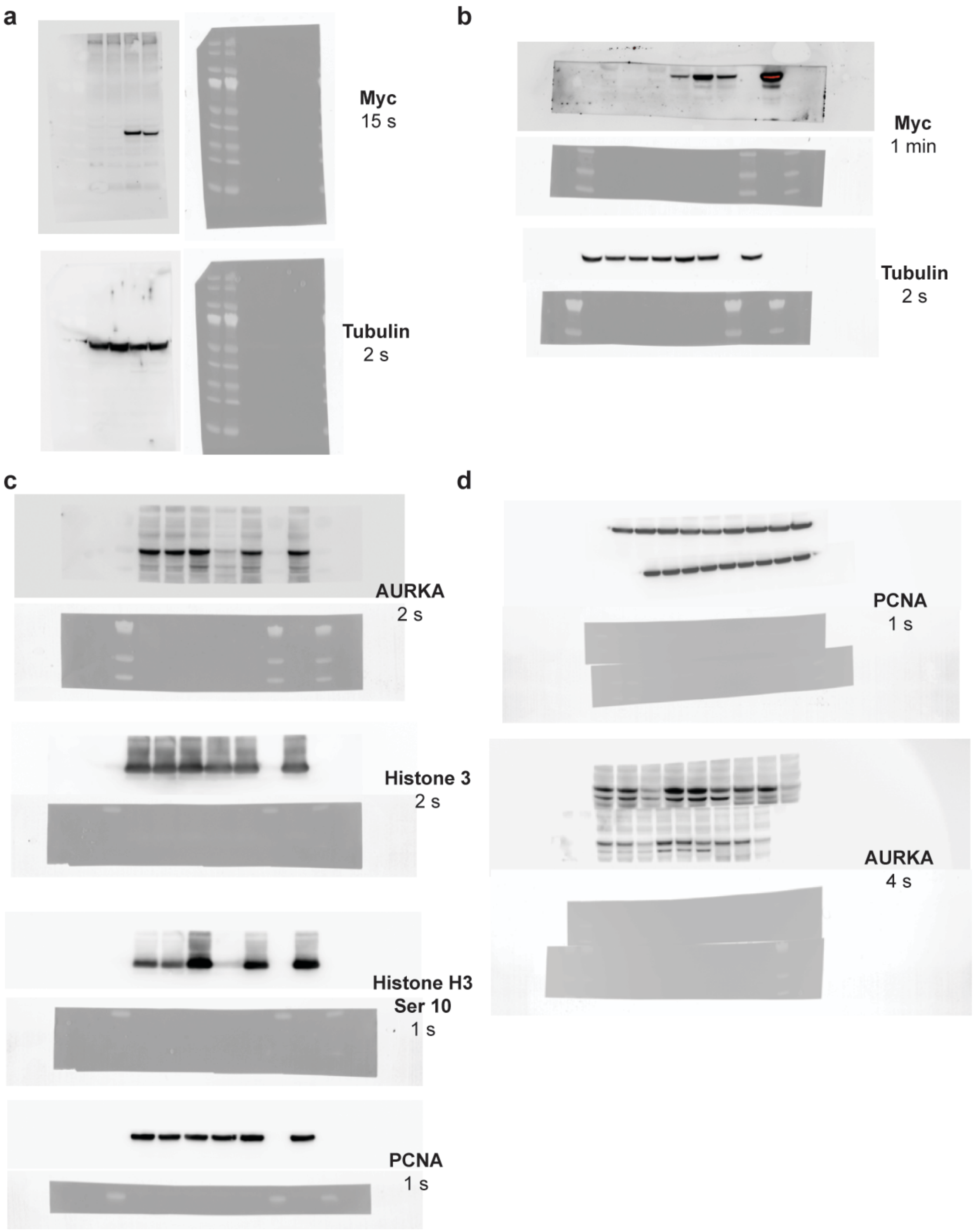

Supplementary Figure 9. Original Western Blot files
Unprocessed immunoblot scans for **a** Myc and Tubulin shown in Figure 2A, **b** Myc and Tubulin in Figure 2B, **c** AURKA, Histone 3, Histone H3 Ser10 and PCNA in Figure 3G, and **d** PCNA and AURKA shown in Figure S5. Exposure times are shown for each blot.

Gene and Chromosomal Location	Gene Expression Pattern[*]		Function	Cancer types with LOH in ≥ 15 % of cases[**]	Estimated number of eligible patients per year[***]		Amino acid change	Functional impact prediction Polyphen-2 (score)	Functional impact prediction SIFT (score)
	Normal tissues	Cancer tissues							
HSD17B4 5q23.1	All (1000-4000).	All tumor types (1000-5000).	Peroxisomal beta-oxidation pathway of fatty acids.	Colon (~17 %) Lung (~21 %) Ovarian (~15 %)	rs25640 (43.95 % HET)	Colon: 49,300 – 54,500 Lung: 79,300 – 87,700 Ovarian: 7400 – 8200	R106H	probably damaging (0.985)	damaging (0.02)
					rs1143650 (3.02 % HET)	Colon: 3,400 – 3,700 Lung: 5,500 – 6,000 Ovarian: 510 – 560	T292S	benign (0.001)	tolerated (0.35)
GSTP1 11q13.2	All (300-8000).	All tumor types (300-5000), especially high in urogenital, lung and cervical cancer.	Conjugation of reduced glutathione to hydrophobic electrophiles.	Melanoma (~16 %) Neuroblastoma (~15 %)	rs1695 (41.67 % HET)	Melanoma: 7,400 – 8,200 Neuroblastoma: 7,600 – 8,400	I105V	benign (0.000)	tolerated (1.00)
					rs1138272 (6.96 % HET)	Melanoma: 1,200 – 1,300 Neuroblastoma: 1,200 – 1,400	A114V	possibly damaging (0.552)	tolerated (0.18)
HAAO 2p21	All (300-1500), especially liver and kidney.	All tumors (200-1200), especially high in GISTs and liver cancers.	Oxidative ring opening of 3-hydroxyanthranilate to 2-amino-3-carboxymuconate semialdehyde.	Ovarian (~16 %)	rs3816182 (39.56 % HET)	Ovarian: 7,100 – 7,900	T42S	benign (0.001)	tolerated (0.99)
					rs3816183 (27.84 % HET)	Ovarian: 5,000 – 5,500	I37V	benign (0.000)	tolerated (1.00)
NAT2 8p22	Restricted to small and large intestine and liver (600-900).	Restricted to CRC and liver cancer (400).	N- or O-acetylation of arylamine and heterocyclic amine substrates.	Colon (~21 %) Lung (~31 %) Ovarian (~22 %) Melanoma (~22 %) Head/neck (~20 %) Kidney (~18 %)	rs1799930 (35.81 % HET)	Colon: 48,600 – 53,700 Lung: 96,200 – 106,300 Ovarian: 9,100 – 10,100 Melanoma: 8,800 – 9,800 Head / neck: 19,400 – 21,400 Kidney: 10,300 – 11,400	R197Q	probably damaging (1.000)	damaging (0.02)
CAPN2 1q41	All (500-2700).	All tumor types (500-2700).	Proteolysis of substrates involved in cytoskeletal remodeling and signal transduction.	Lung (~18 %) Ovarian (~17 %)	rs17599 (32.60 % HET)	Lung: 51,700 – 57,100 Ovarian: 6,400 – 7,100	K568Q	benign (0.007)	damaging (0.02)
					rs9804140 (10.90 % HET)	Lung: 17,200 – 19,100 Ovarian: 2,100 – 2,300	K476R	benign (0.001)	tolerated (1.00)
ADPRHL2 1p34.3	All(700-2700).	All tumor types (700-2000).	Poly(ADP-ribose) metabolism, required for maintenance of the normal function of neuronal cells.	Colon (~17 %) Lung (~27 %) Breast (~19 %) Ovarian (~20 %) Melanoma (~18 %) Neuroblastoma (~52 %)	rs2236387 (26.19 % HET)	Colon: 29,600 – 32,700 Lung: 62,200 – 68,700 Breast: 39,600 – 43,800 Ovarian: 5,900 – 6,500 Melanoma: 5,400 – 5,900	E209K	benign (0.000)	damaging (N/A)

						Neuroblastoma: 16,500 – 18,300			
CTSC 11q14.2	All (300-5500) except neural tissues and testis.	All tumor types (300-5500) except brain tumors and prostate cancer.	Thiol protease with dipeptidylpeptidase activity.	Melanoma (~15 %) Neuroblastoma (~15 %)	rs3888798 (16.30 % HET)	Melanoma: 2,600 – 2,900 Neuroblastoma: 2,900 – 3,200	I453V	benign (0.444)	tolerated (0.07)
DUSP23 1q23.2	All (300-1400) except neural tissues.	All tumor types (400-1500) except brain tumors.	Protein phosphatase mediating dephosphorylation of proteins on Tyr and Ser/Thr residues.	Lung (~15 %)	rs1129923 (10.16 % HET)	Lung: 13,800 – 15,200	G131S	probably damaging (1.000)	damaging (N/A)
AKR7A2 1p36.13	All (500-2700).	All tumor types (500-2000).	NADPH-dependent reduction of succinic semialdehyde to gamma-hydroxybutyrate.	Colon (~20 %) Lung (~28 %) Breast (~22 %) Ovarian (~25 %) Melanoma (~18 %) Neuroblastoma (~52 %)	rs1043657 (8.97 % HET)	Colon: 11,600 – 12,800 Lung: 22,300 – 24,600 Breast: 16,200 – 18,000 Ovarian: 2,500 – 2,800 Melanoma: 1,800 – 2,000 Neuroblastoma: 5,600 – 6,200	A142T	probably damaging (0.998)	damaging (0.04)
					rs2231203 (4.76 % HET)	Colon: 6,100 – 6,800 Lung: 11,800 – 13,000 Breast: 8,600 – 9,500 Ovarian: 1,300 – 1,400 Melanoma: 980 – 1,000 Neuroblastoma: 3,000 – 3,300	S255N	benign (0.000)	tolerated (0.40)
NQO2 6p25.2	All (300-3200).	All tumor types (300-2000).	Quinone reductase involved in detoxification pathways and vitamin K-dependent gamma-carboxylation of glutamate residues.	Lung (~18 %) Ovary (~15 %)	rs28383623 (6.96 % HET)	Lung: 11,000 – 12,200 Ovarian: 1,100 – 1,300	K16R	benign (0.000)	tolerated (0.47)
PNPO 17q21.32	All (400-2200), especially liver.	All tumor types (300-1700), especially liver cancer.	Oxidation of pyridoxine 5'-phosphate (PNP) a pyridoxamine 5'-phosphate (PMP) into pyridoxal 5'-phosphate (PLP).	Lung (~17 %) Ovarian (~21 %)	rs17679445 (6.14 % HET)	Lung: 9,000 – 9,900 Ovarian: 1,400 – 1,600	R116Q	probably damaging (0.974)	tolerated (0.41)
SULT1A1 16p11.2	All (500-5000).	All tumor types (500-3000).	Sulfate conjugation of catecholamines, phenolic drugs, estrogen and neurotransmitters.	Lung (~19 %) Ovarian (~16 %) Melanoma (~21 %)	rs28374453 (5.13 % HET)	Lung: 8,700 – 9,600 Ovarian: 940 – 1,000 Melanoma: 1,200 – 1,300	F247L	N/A	tolerated (0.13)

AKR1A1<br><br>1p34.1	All tissues (800-5000).	All tumor types (1000-3000).	NADPH-dependent reduction of aromatic and aliphatic aldehydes. Reduction of mevaldate to mevalonic acid and of glyceraldehyde to glycerol. Broad substrate specificity.	Colon (~18 %)<br>Lung (~27 %)<br>Breast (~19 %)<br>Ovarian (~20 %)<br>Melanoma (~18 %)<br>Neuroblastoma (~52 %)	rs2229540 (5.13 % HET)	Colon: 6,200 – 6,800<br>Lung: 12,100 – 13,400<br>Breast: 7,800 – 8,600<br>Ovarian: 1,100 – 1,200<br>Melanoma: 1,000 – 1,100<br>Neuroblastoma : 3,200 – 3,600	N52S	probably damaging (1.000)	tolerated (0.52)

Supplementary Table 1. Target genes for loss of heterozygosity-based therapeutic approaches in common cancer

Twenty-five genes with prevalent nsSNVs near active sites of enzymes were selected according to gene expression (*23*) (normalized expression values ≥ 300 in specified tissues) and LOH in common cancers (breast, colon, kidney, lung, ovarian, head and neck, malignant melanoma and neuroblastoma (*18*), resulting in 13 genes expressed in at least one of the 8 tumor types also having LOH in ≥ 15% of cases. Genes are sorted by highest to lowest heterozygosity frequency for the filtered SNVs. The functional impact of the SNVs was predicted using the Polyphen-2 and SIFT algorithms.

Gene	SNV	Genotype	Reference AAF	Frequency (*n*, %)		LOH in tumor			HWE
				Normal	Tumor	Total	Wild-type	Variant	
NAT2	rs1799930	G/G	58.8	41 (55.4)	44 (59.5)	5 (6.8)	2	3	0.169
		G/A	35.2	31 (41.9)	26 (35.1)				
		A/A	6.4	2 (2.7)	4 (5.4)				
	rs1801280	T/T	50.6	18 (24.3)	21 (28.4)	7 (9.5)	4	3	0.245
		T/C	34.8	39 (52.7)	32 (43.2)				
		C/C	18.5	17 (23.0)	21 (28.4)				
ABP1	rs1049793	C/C	34.9	32 (43.2)	34 (46.0)	2 (2.7)	0	2	0.903
		C/G	47.1	35 (47.3)	33 (44.6)				
		G/G	19	9 (12.2)	9 (12.2)				
AKR7A2	rs1043657	G/G	89.1	61 (82.4)	63 (85.1)	2 (2.7)	0	2	0.407
		G/A	12.9	13 (17.6)	11 (14.9)				
		A/A	2.7	0	0				
SULT1C3	rs2219078	G/G	41.9	48 (64.9)	49 (66.2)	1 (1.4)	0	1	0.907
		G/A	40	23 (31.1)	22 (29.7)				
		A/A	19	3 (4.1)	3 (4.1)				
SULT1A1	rs9282861	G/G	61.3	41 (55.4)	41 (55.4)	0	0	0	0.014
		G/A	32.7	33 (44.6)	33 (44.6)				
		A/A	7.3	0	0				

Supplementary Table 2. Loss of heterozygosity at prevalent catalytic site SNVs in colorectal cancers

Genomic DNA from 74 chromosomally unstable CRCs and corresponding patient-matched normal tissues were genotyped by PCR coupled Sanger sequencing. The genotype frequencies were compared to the average allele frequency extracted from the 1000 Genomes database (AAF). Hardy-Weinberg equilibrium (HWE) was calculated in normal samples.

Plate	Well	CBCS compound ID	SMILES string	Structure
1	A01	CBK001266	COC1=CC=CC=C1NS(=O)(=O)C1=CC=C(N2CCN3CCCC3C2)C(N)=C1	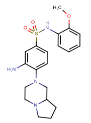
1	B01	CBK015754	NC1=CC=C(C#N)C2=C1C=CC=C2	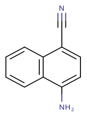
1	C01	CBK066880	CC1=NC2=C(C)C=C(Br)C(N)=C2C(=C1)N1CCOCC1	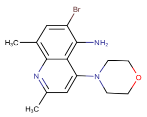
1	D01	CBK036728	COC1=CC=C(C=C1)C1=C(N=CC(=C1)C(=O)NCC1=CC=C(N)C=C1)C1=CC=CC(C)=C1	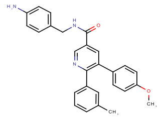
1	E01	CBK065642	COC1=CC(NC2=CN=CC(=N2)C2=CC=C(N)C=C2)=CC(OC)=C1OC	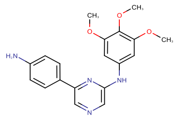
1	F01	CBK037221	NC1=CC(=CC=C1)C1=CN=CC(NCC2CCOCC2)=N1	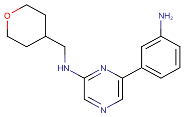
1	G01	CBK093729	CC(C)NC1=CC(SC2=C(N)C=CC=C2)=NC=N1	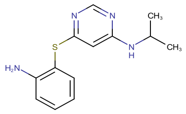
1	H01	CBK041657C	NC1=C(Br)C=C(Br)C=C1CNC1CCC(O)CC1	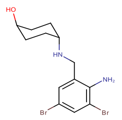
1	A02	CBK157034	CCCCCCOC(=O)C1=CC=C2C(=O)N(C(=O)C2=C1)C1=CC=CC(N)=C1	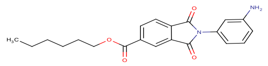
1	B02	CBK084324	NC1=CC=C(C2=CC(=CC=C2)C(F)(F)F)C2=CC=CC=C12	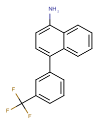
1	C02	CBK091099	NC1=CC=C(C=C1)C(=O)NC1=CC=C(NC2=CC(=NC=N2)C2=CC=CO2)C=C1	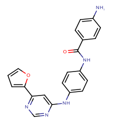

1	D02	CBK126908	CC1=NC2=C(C(N)=C(C)C=C2C)C(=C1)N1CCOCC1	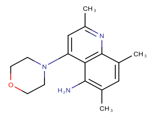
1	E02	CBK001518C	[H]N(C1=CC=C(C=C1)C(F)(F)F)S(=O)(=O)C1=CC(N)=C(C=C1)N1CCCN([H])CC1	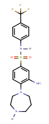
1	F02	CBK085854	NC1=C(SC2=NC=CC=C2)C=CC=C1	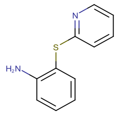
1	G02	CBK036655	CC1=CC(=CC=C1)C1=NC=C(NC(=O)CCOC2=CC=CC=C2)N=C1C1=CC=C(N)C=C1	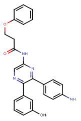
1	H02	CBK036985	NC1=CC(=CC=C1)C1=CN=C(NCC2=CC(F)=C(F)C(F)=C2)C=C1	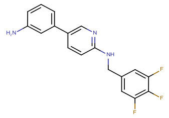
1	A03	CBK065658	CCNC1=CN=CC(=N1)C1=CC=C(N)C=C1	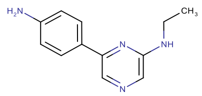
1	B03	CBK065270	CN(C)CCN(C)C1=NC(=CN=C1)C1=CC=CC(N)=C1	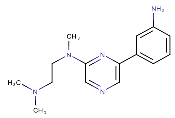
1	C03	CBK037580	NC1=CC=C(CCNC(=O)C2=CC(C3=CC=NC=C3)=C(N=C2)C2=CC=CC(=C2)C(F)(F)F)C=C1	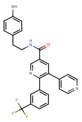
1	D03	CBK065751	CCN(CC)CCN1CCN(CC1)C1=CN=CC(=N1)C1=CC=CC(N)=C1	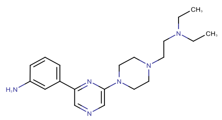
1	E03	CBK074823	NC1=C(SC(CC(=O)C2=CC=CC=C2)C2=CC3=C(OCO3)C=C2)C=CC=C1	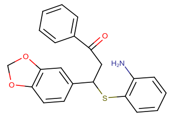
1	F03	CBK084327	NC1=CC=C(C2=CC=C(OC(F)(F)F)C=C2)C2=CC=CC=C12	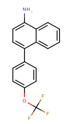
1	G03	CBK091103	CC(=O)NC1=CC=C(C=C1)C1=NC=NC(NC2=CC=C(NC(=O)C3=CC=C(N)C=C3)C=C2)=C1	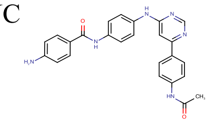
1	H03	CBK061872	CC(C)(C)C1=CC=C(C=C1)C1=NC2=NC(N)=CC=C2C=C1	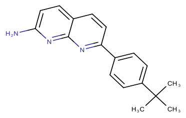

1	A04	CBK001976C	NC1=CC=CC2=C(OCCO)C=CC=C12	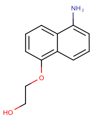
1	B04	CBK015997	NC1=CC=C2N=CN=C3C4=C(C=CC=C4)C(=O)C1=C23	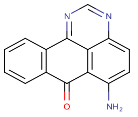
1	C04	CBK038080	CC1=CC=CC(CNC2=NC=CN=C2C2=CC=CC(N)=C2)=C1	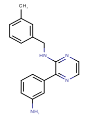
1	D04	CBK037561	COC1=CC=C(C=C1)C1=C(N=CC(=C1)C(=O)NCCC1=CC=C(N)C=C1)C1=CC=C(Cl)C=C1	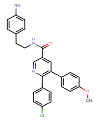
1	E04	CBK065681	NC1=CC=C(C=C1)C1=NC(=CN=C1)N1CCN(CC1)C1=CC=NC=C1	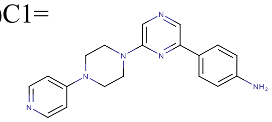
1	F04	CBK065273	[H][C@](C)(NC1=NC(=CN=C1)C1=CC=CC(N)=C1)C1=CC=CC=C1	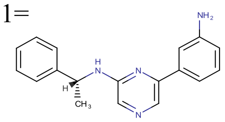
1	G04	CBK063954	CCCC\C=C\C1=NC(=C(N)N=C1)C1=CC(N)=CC=C1	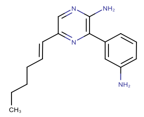
1	H04	CBK066592	NC1=CC=C(C=C1)C1=CN2C=CN=C2C(NCC2=CC=CC=C2)=N1	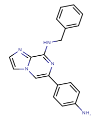
1	A05	CBK083570	NC1=CC=C(C=C1)C(=O)OC1=CC=C(OC2=CC=CC=C2)C=C1	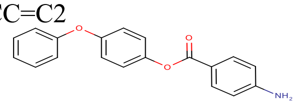
1	B05	CBK084332	COC1=CC(=CC(OC)=C1OC)C1=CC=C(N)C2=CC=CC=C12	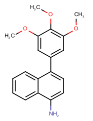
1	C05	CBK091434	CC(C)NC1=NC(SC2=CC(N)=CC=C2)=CN=C1	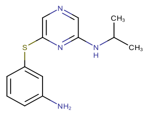
1	D05	CBK126959T	NC1=CC=CC(=C1)C1=CC=C(OC2CCNCC2)C=C1	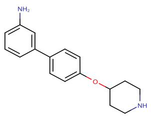
1	E05	CBK084326	CS(=O)(=O)C1=CC=C(C=C1)C1=CC=C(N)C2=CC=CC=C12	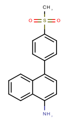

1	F05	CBK017167	NC1=CC2=C(NC(=N2)C2=NC=CC=C2)C=C1	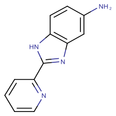
1	G05	CBK036684	CC1=CC(=CC=C1)C1=NC=C(NC(=O)CCC2=CN=CC=C2)N=C1C1=CC=C(N)C=C1	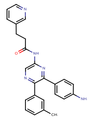
1	H05	CBK037619	COC(=O)C1=C(OC)C=C(NC2=NC(=CC=C2)C2=CC=CC(N)=C2)C=C1	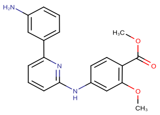
1	A06	CBK065808	NC1=CC(=CC=C1)C1=CN=CC(=N1)N(CCO)CCO	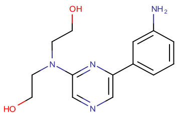
1	B06	CBK065278	CCC(CO)NC1=NC(=CN=C1)C1=CC=CC(N)=C1	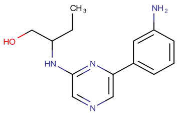
1	C06	CBK065265	NC1=CC(=CC=C1)C1=CN=CC(NC2=CC=CC=C2)=N1	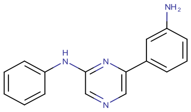
1	D06	CBK091091	NC1=CC=C(C=C1)C(=O)NC1=CC=C(NC2=CC(=NC=N2)C2=CC(=CC(=C2)C(F)(F)F)C(F)(F)F)C=C1	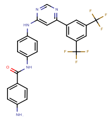
1	E06	CBK093776	NC1=C(SC2=NC3=CC=CC=C3N=C2C(F)(F)F)C=CC=C1	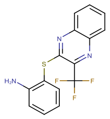
1	F06	CBK085844	NC1=CC=CC(SC2=NC=CC=N2)=C1	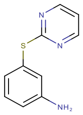
1	G06	CBK093758	NC1=C(SC2=NC=CC=C2C(F)(F)F)C=CC=C1	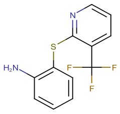
1	H06	CBK081218	NC1=CC2=CC=CN=C2C(=N1)C1=CSC=C1	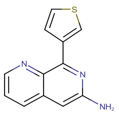
1	A07	CBK006263	NC1=C(SC2=NC(=CC=N2)C(F)(F)F)C=CC=C1	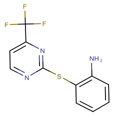
1	B07	CBK081199	CC(C)(C)C1=CC=C(C=C1)C1=CC2=NC=CC=C2N=C1N	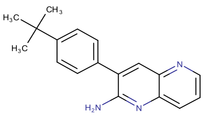

1	C07	CBK037512	NC1=CC(=CC=C1)C1=NC=CN=C1NCCC1=CC=CC=N1	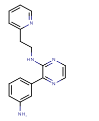
1	D07	CBK037823	NC1=CC=C(C=C1)C(=O)NC1=CC=C(NC2=CC(=NC=N 2)C2=CC=CN=C2)C=C1	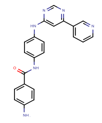
1	E07	CBK036722	CC(=O)C1=CC=C(OCC(=O)NC2=CN=C(C3=CC=CC(C) =C3)C(=N2)C2=CC=C(N)C=C2)C=C1	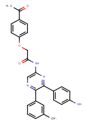
1	F07	CBK066453	NC1=CC=C(C=C1)C1=CN2C=CN=C2C(NC2=CC=C(Cl) C=C2)=N1	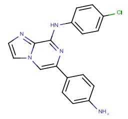
1	G07	CBK037583	CC1=CC(=CC=C1)C1=NC=C(NC(=O)CC2=CC(OC3=C C=CC=C3)=CC=C2)N=C1C1=CC=C(N)C=C1	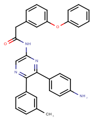
1	H07	CBK091092	NC1=CC=C(C=C1)C(=O)NC1=CC=C(NC2=CC(=NC=N 2)C2=C(OCC3=CC=CC=C3)C=CC=C2)C=C1	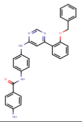
1	A08	CBK084312	NC1=CC=C(C2=CC=CC=C2)C2=CC=CC=C12	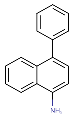
1	B08	CBK087401	COC1=C(C(=O)C2=C(O1)C=C(N)C=C2)C1=CC=C(C)C =C1	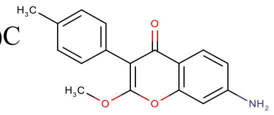
1	C08	CBK093761	NC1=C(SC2=NC=CC(=C2)C(F)(F)F)C=CC=C1	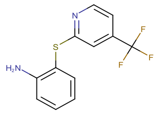
1	D08	CBK200102T	COC1=CC=C(C=C1)C(C)C1=CC2=C(OCO2)C=C1N	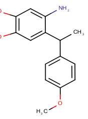
1	E08	CBK010155T	NC1=CC2=C(NC(=O)\C2=C/C2=CNC3=C2C=CC=N3)C =C1	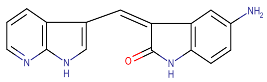
1	F08	CBK067622	NC1=NC2=NC(=CC=C2C=C1)C1=CC=NC=C1	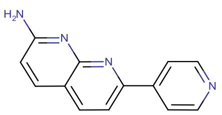
1	G08	CBK089310	NC1=CC(=CC=C1)C1=NC=CN=C1NCC1=CN=CC=C1	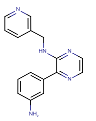

1	H08	CBK036694	CC1=CC(=CC=C1)C1=NC=C(NC(=O)C2=CC=CC=N2)N=C1C1=CC=C(N)C=C1	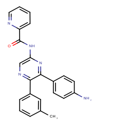
1	A09	CBK037463	NC1=CC=C(NC(=O)C2=CC=C(NC(=O)C3=CC(C4=CC=NC=C4)=C(N=C3)C3=CC=CC=C3)C=C2)C=C1	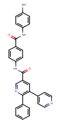
1	B09	CBK037236	COC1=CC(NC2=CC(=NC3=CC=NN23)C2=CC=CC(N)=C2)=CC(OC)=C1OC	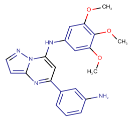
1	C09	CBK038032	COC1=CC(=CC=C1O)C(O)CNC(=O)C1=CC(C2=CC=CC(N)=C2)=C(N=C1)C1=CC=C(F)C=C1	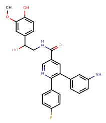
1	D09	CBK066564	NC1=CC=C(C=C1)C1=CN2C=CN=C2C(NCC2=CC=CS2)=N1	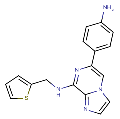
1	E09	CBK084314	NC1=CC=C(C2=CC(F)=CC(F)=C2)C2=CC=CC=C12	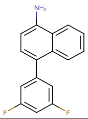
1	F09	CBK087552	NC1=C2C=CC=C3C(=O)N(C4=CC=CC=C4)C(=O)C(C=C1)=C23	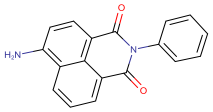
1	G09	CBK093782	NC1=C(SC2=CC=C(C=N2)C(F)(F)F)C=CC=C1	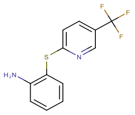
1	H09	CBK158569	NC1=NC=C(C=C1C(=O)NCC1=C(F)C=CC=C1F)C1=CC=C(C=C1)C(=O)N1CCOCC1	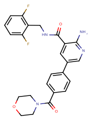
1	A10	CBK010324	[H]N([H])C1=CC=C(C=C1)S(=O)(=O)NC1=NN=C(OC(C)C)S1	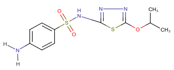
1	B10	CBK017557	[H]N([H])C1=CC2=C(C=C1)C(O)=C(C(=O)NCC1=CC=C(Cl)C=C1)C(=O)N2	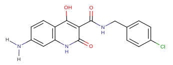
1	C10	CBK036686	NC1=CC=C(CCNC(=O)C2=CC(C3=CC=CO3)=C(N=C2)C2=CC=C(Cl)C=C2)C=C1	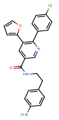
1	D10	CBK036749	CC1=CC(=CC=C1)C1=NC=C(NC(=O)CC2OC(=O)C3=C2C=CC=C3)N=C1C1=CC=C(N)C=C1	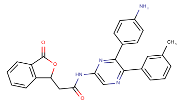

1	E10	CBK036786	NC1=CC(=CC=C1)C1=C(N=CC=N1)N1CCCC1CO	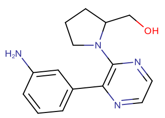
1	F10	CBK063906	NC1=CC=CC(=C1)C1=C(N)N=CC(=N1)C1=CN=CC=C1	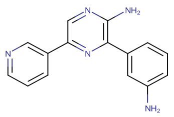
1	G10	CBK037600	CC1=CC=C(C=C1)S(=O)(=O)NCC(=O)NC1=CN=C(C2=CC=CC(C)=C2)C(=N1)C1=CC=C(N)C=C1	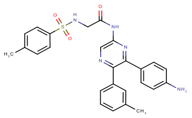
1	H10	CBK066677	NC1=CC=C(C=C1)C1=CN2C=CN=C2C(NCC2CC2)=N1	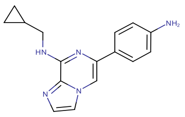
1	A11	CBK084325	NC1=CC=C(C2=CC=C3OCCCOC3=C2)C2=CC=CC=C12	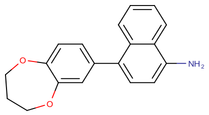
1	B11	CBK087802	COC(=O)C1=CC(=CC(=C1)C(=O)OC)S(=O)(=O)NC1=CC(N)=C(Cl)C=C1	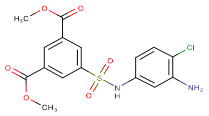
1	C11	CBK111303	COC1=CC2=C(OC=C2C(=O)C2=CC=C(N)C=C2)C=C1	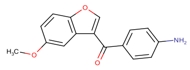
1	D11	CBK067625	NC1=NC2=NC(=CC=C2C=C1)C1=CC2=C(OCC2)C=C1	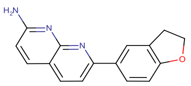
1	E11	CBK011865C	CNC1=NC(NC)=NC(NS(=O)(=O)C2=CC=C(N)C=C2)=C1	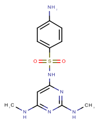
1	F11	CBK024211C	COC1=C(OCCN2C=CN=C(N3CCNCC3)C2=O)C=C(N)C=C1	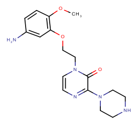
1	G11	CBK036690	NC1=CC=C(C=C1)C1=NC(NC(=O)CC2=CC=CC=C2)=CN=C1C1=CC(Cl)=C(F)C=C1	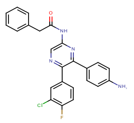
1	H11	CBK065578	NC1=CC=C(C=C1)C1=NC(NCC2=CC=CC=C2)=CN=C1	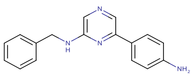
2	A01	CBK037049	NC1=CC(=CC=C1)C1=NC2=CC=NN2C(=C1)N1CCCC1CO	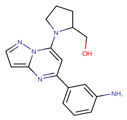

2	B01	CBK037537	NC1=CC(=CC=C1)C1=NC=CN=C1NCC1=CC=CS1	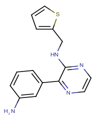
2	C01	CBK093726	CC(C)NC1=NC(SC2=C(N)C=CC=C2)=CN=C1	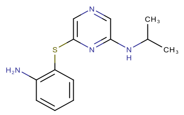
2	D01	CBK074456	COC1=C(OC)C=C(C=C1)C1=CSC(NC2=CC=C(N)C=C2)=N1	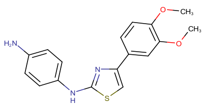
2	E01	CBK084319	COC1=C(C=CC(F)=C1)C1=CC=C(N)C2=CC=CC=C12	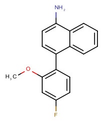
2	F01	CBK089485	CC(C)NC1=NC=CN=C1SC1=CC=C(N)C=C1	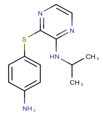
2	G01	CBK115239	NC1=CC=CC(SC2=CC=CC(=N2)C(F)(F)F)=C1	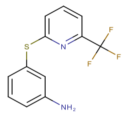
2	H01	CBK067624	NC1=NC2=NC(=CC=C2C=C1)C1=CSC=C1	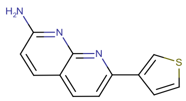
2	A02	CBK041544	NC(=N)NS(=O)(=O)C1=CC=C(N)C=C1	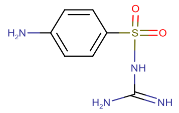
2	B02	CBK201096	CC(=O)N([Na])S(=O)(=O)C1=CC=C(N)C=C1	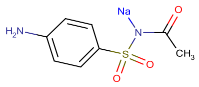
2	C02	CBK015484	NC1=CC=C(C=C1)S(=O)(=O)NC1=NC=CS1	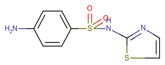
2	D02	CBK016845	NC1=CC=C(C=C1)S(=O)(=O)NC1=CC=NN1C1=CC=CC=C1	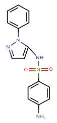
2	E02	CBK041380	NC1=CC=C(C=C1)S(=O)(=O)NC1=NC=CC=N1	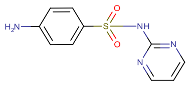
2	F02	CBK011640	NC1=CC=C(C=C1)S(=O)(=O)C1=CC=C(N)C=C1	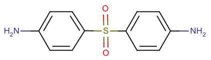

2	G02	CBK004330C	CCN(CC)CCOC(=O)C1=CC=C(N)C=C1	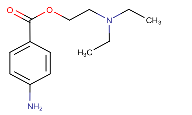
2	H02	CBK200750C	CCCCOC1=CC(=CC=C1N)C(=O)OCCN(CC)CC	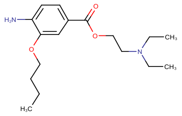
2	A03	CBK041709G	CN1CC(C2=CC=CC=C2)C2=CC=CC(N)=C2C1	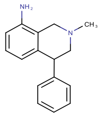
2	B03	CBK015559	CC1=CC(NS(=O)(=O)C2=CC=C(N)C=C2)=NO1	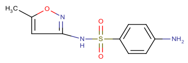
2	C03	CBK023827C	CCN(CC)CCNC(=O)C1=CC(Cl)=C(N)C=C1OC	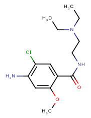
2	D03	CBK042127	CCC1(CCC(=O)NC1=O)C1=CC=C(N)C=C1	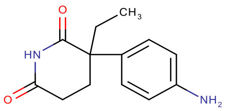
2	E03	CBK041395	CC1=NOC(NS(=O)(=O)C2=CC=C(N)C=C2)=C1C	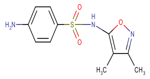
2	F03	CBK011723C	CCN(CC)CCNC(=O)C1=CC=C(N)C=C1	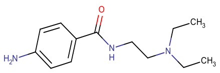
2	G03	CBK041771C	CC(C)(C)NCC(O)C1=CC(Cl)=C(N)C(Cl)=C1	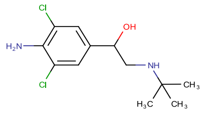
2	H03	CBK200689C	NC1=C(Br)C=C(Br)C=C1CN[C@H]1CC[C@H](O)CC1	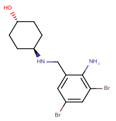
2	A04	CBK041870G	COC1=C(C=C(Cl)C(N)=C1)C(=O)NC1CCN(CC2=CC=CC=C2)CC1	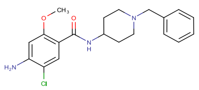
2	B04	CBK041324N	COC1=CC(=CC=C1\N=N\C1=C(O)C2=C(C=C1)C(=CC(=C2N)S([O-])(=O)=O)S([O-])(=O)=O)C1=CC(OC)=C(C=C1)\N=N\C1=C(O)C2=C(N)C(=CC(=C2C=C1)S([O-])(=O)=O)S([O-])(=O)=O	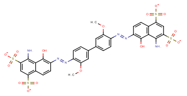
2	C04	CBK200709	CO[C@@H]1CN(CCCOC2=CC=C(F)C=C2)CC[C@@H]1NC(=O)C1=CC(Cl)=C(N)C=C1OC	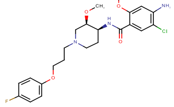

2	D04	CBK200806	NC1=C(C=C(C(=C1)C(Cl)=C(Cl)Cl)S(N)(=O)=O)S(N)(=O)=O	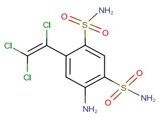
2	E04	CBK200956	CCN1CCCC1CNC(=O)C1=C(OC)C=C(N)C(=C1)S(=O)(=O)CC	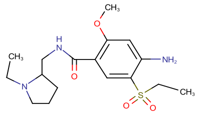
2	F04	CBK041664	CCN(CC)CCNC(=O)C1=CC(Br)=C(N)C=C1OC	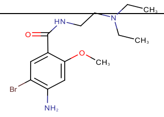
2	G04	CBK041378	NC1=CC=C(C=C1)S(=O)(=O)NC(=O)C1=CC=CC=C1	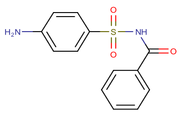
2	H04	CBK042111	CCOC(=O)C1=CC=C(N)C=C1	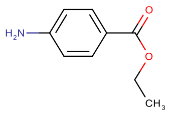
2	A05	CBK041541	NC1=CC=C(C=C1)S(=O)(=O)NC1=CC=C(Cl)N=N1	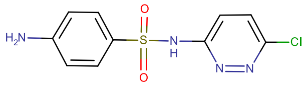
2	B05	CBK015623	COC1=CC=C(NS(=O)(=O)C2=CC=C(N)C=C2)N=N1	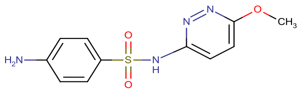
2	C05	CBK041542	COC1=CC(NS(=O)(=O)C2=CC=C(N)C=C2)=NC(OC)=N1	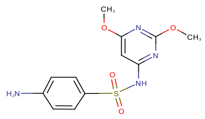
2	D05	CBK004272	NC1=CC=C(C=C1)S(N)(=O)=O	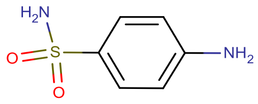
2	E05	CBK041543N	NC1=CC=C(C=C1)S(=O)(=O)[N-]C1=NC2=CC=CC=C2N=C1	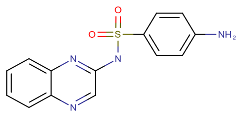
2	F05	CBK026275	CC1=NN=C(NS(=O)(=O)C2=CC=C(N)C=C2)S1	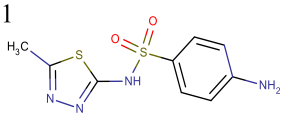
2	G05	CBK042138	CCCCOC(=O)C1=CC=C(N)C=C1	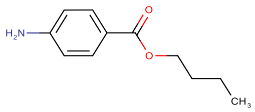
2	H05	CBK016686	NC1=CC=C(C=C1)S(=O)(=O)NC1=NC=CC=C1	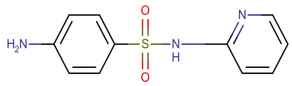

2	A06	CBK015675	COC1=CN=C(NS(=O)(=O)C2=CC=C(N)C=C2)N=C1	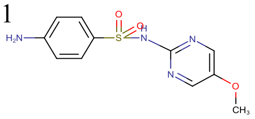
2	B06	CBK200778N	CC1=CC(C)=NC([N-]S(=O)(=O)C2=CC=C(N)C=C2)=N1	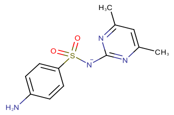
2	C06	CBK201114I	CC[N+](C)(CC)CCC[N+]1=C(C2=CC=CC=C2)C2=C(C=CC(N)=C2)C2=C1C=C(N)C=C2	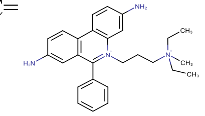
2	D06	CBK041545	COC1=CC(NS(=O)(=O)C2=CC=C(N)C=C2)=NC=N1	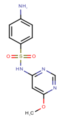
2	E06	CBK041769	CCCCN(CCCC)CCCOC(=O)C1=CC=C(N)C=C1	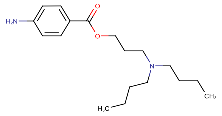
2	F06	CBK200816	CC(CN(C(C)=O)C1=C(I)C=C(I)C(N)=C1I)C(O)=O	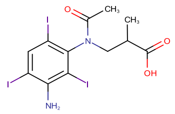
2	G06	CBK004349	NC1=CC=C(C=C1)C(=O)NCC(O)=O	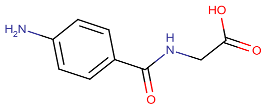
2	H06	CBK200830	NC1=CC=CC=C1S(N)(=O)=O	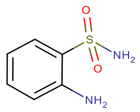
2	A07	CBK200907C	CCCOC1=C(N)C=C(C=C1)C(=O)OCCN(CC)CC	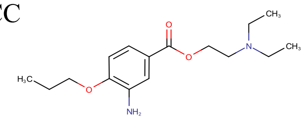
2	B07	CBK041777	CCC(CC1=C(I)C=C(I)C(N)=C1I)C(O)=O	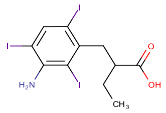
2	C07	CBK200909C	CCCOC1=C(C=CC(N)=C1)C(=O)OCCN(CC)CC	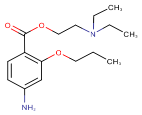
2	D07	CBK200885	NC1=CC(C(O)=O)=C(O)C=C1	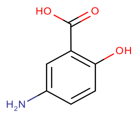
2	E07	CBK200918	COC1=NC=NC(NS(=O)(=O)C2=CC=C(N)C=C2)=C1OC	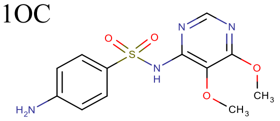

2	F07	CBK041721	NC1=CC(O)=C(C=C1)C(O)=O	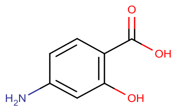
2	G07	CBK027966C	CN(CC1=CC(Br)=CC(Br)=C1N)C1CCCCC1	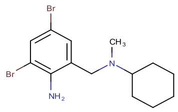
2	H07	CBK201006	C[C@H]1CN(C[C@@H](C)N1)C1=C(F)C2=C(C(N)=C1F)C(=O)C(=CN2C1CC1)C(O)=O	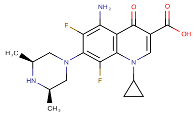
2	A08	CBK200505	CC1=CC=NC(NS(=O)(=O)C2=CC=C(N)C=C2)=N1	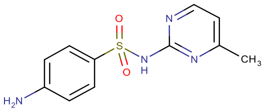
2	B08	CBK020177	COC1=CC(N)=CC=C1	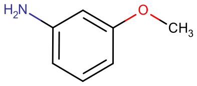
2	C08	CBK066126	COC1=C(OC)C=C(C=C1)N1CCN(CC1)C1=CN=CC(=N1)C1=CC=CC(N)=C1	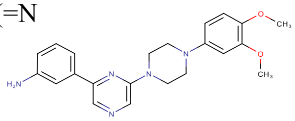
2	D08	CBK041438C	C[N+]1=C2C=C(N)C=CC2=CC2=CC=C(N)C=C12	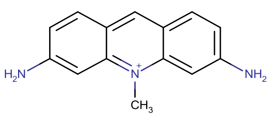
2	E08	CBK041754B	CC[N+]1=C(C2=CC=CC=C2)C2=CC(N)=CC=C2C2=CC=C(N)C=C12	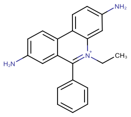
2	F08	CBK038016	NC1=CC(=CC=C1)C1=C(N=CC(=C1)C(=O)NCCC1=CN=CC=C1)C1=CC=C(F)C=C1	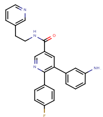
2	G08	CBK036783	CCOC1=C(C=CC=C1)C(=O)NC1=CN=C(C2=CC=CC(C)=C2)C(=N1)C1=CC=C(N)C=C1	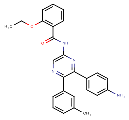
2	H08	CBK090766	NC1=CC(=CC=C1)C1=NC=NC(NC2=CC=C(CCO)C=C2)=C1	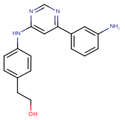
2	A09	CBK090886	NC1=CC(=CC=C1)C1=NC=NC(NC2=CC=C(Cl)C=C2)=C1	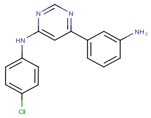
2	B09	CBK090915	NC1=CC(=CC=C1)C1=NC=NC(NC2=CC(OCC3=CC=CC=C3)=CC=C2)=C1	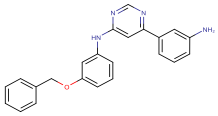

2	C09	CBK091145	CC1=CC=C(NC2=CC(=NC=N2)C2=CC=CC(N)=C2)C=C1NS(C)(=O)=O	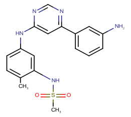
2	D09	CBK091187	NC1=CC(=CC=C1)C1=NC=NC(NC2=CC=C(CC#N)C=C2)=C1	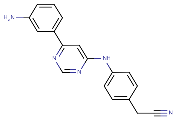
2	E09	CBK063585	COC1=CC=C(NC2=C(N)N=CC(=N2)C2=CC(N)=CC=C2)C=C1	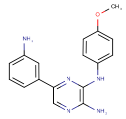
2	F09	CBK018077C	NC1=CC(=CC=C1S(=O)(=O)C1=CC=CC(F)=C1)N1CCCNCC1	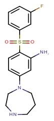
2	G09	CBK016709	CCC(C1=CC=C(N)C=C1)C1=C(O)C2=C(OC1=O)C=CC=C2	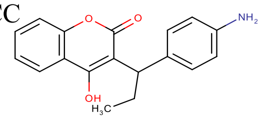
2	H09	CBK063930	NC1=CC=CC(=C1)C1=NC(=CN=C1N)C1=CC=C(Cl)C=C1	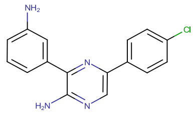
2	A10	CBK091191	NC1=CC(=CC=C1)C1=NC=NC(NC2=CC=C(C=C2)C2CCCCC2)=C1	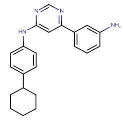
2	B10	CBK063915	NC1=CC=CC(=C1)C1=NC(=CN=C1N)C1=C(F)C=C(F)C=C1	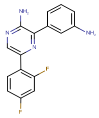
2	C10	CBK018144	NC1=C(C=C2C(=O)NC(=O)C(C#N)=C2N1)C(=O)C1=CC=CC=C1	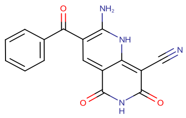
2	D10	CBK061916	CS(=O)(=O)C1=CC=C(C=C1)C1=CN=C2N=C(N)C=CC2=C1	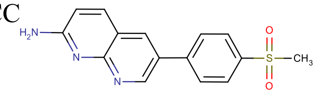
2	E10	CBK081200	NC1=NC2=CC=CN=C2C=C1C1=CC(=CC=C1)C(F)(F)F	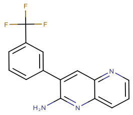
2	F10	CBK081198	CCCCC1=CC=C(C=C1)C1=CC2=NC=CC=C2N=C1N	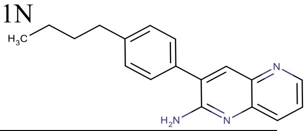
2	G10	CBK158577	NC1=NC=C(C(=C1C(=O)NCCC1=CC=C(O)C=C1)C1=CC=C(C=C1)C(=O)N1CCOCC1	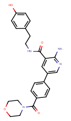

2	H10	CBK160205	CC(=O)N1CCC2=CC=C(NC(=O)C3=CC(=CN=C3N)C3=CC=C(C=C3)C(=O)N3CCOCC3)C=C12	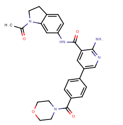
2	A11	CBK160204	NC1=NC=C(C=C1C(=O)NC1=CC=C(C=C1)C(F)(F)F)C1=CC=C(C=C1)C(=O)N1CCOCC1	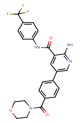
2	B11	CBK061908	NC1=NC(C2=CC(F)=CC(F)=C2)=C2N=CC=CC2=C1	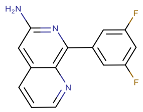
2	C11	CBK061881	NC1=NC2=NC=C(C=C2C=C1)C1=CC=CC=C1	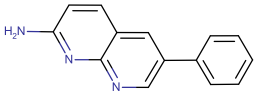
2	D11	CBK158568	CSCCCNC(=O)C1=CC(=CN=C1N)C1=CC=C(C=C1)C(=O)N1CCOCC1	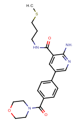
2	E11	CBK158599	CS(=O)(=O)C1=CC=C(CNC(=O)C2=CC(=CN=C2N)C2=CC=C(C=C2)C(=O)N2CCOCC2)C=C1	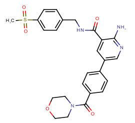
2	F11	CBK081214	NC1=NC2=CC=CN=C2C=C1C1=CSC=C1	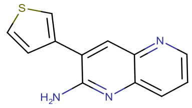
2	G11	CBK067648	CS(=O)(=O)C1=CC=C(C=C1)C1=C2C=CC(N)=NC2=NC=C1	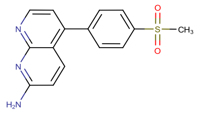
2	H11	CBK100822	CCOC(=O)C1=CC2=C(N=C1N)N(CC)C1=C2C=CC=C1	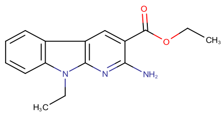

Supplementary Table 3. The set of potential NAT2 substrates used for differential cytotoxicity compound screening.

Protein name	APA (POC)	NAPA (POC)	APA binding affinity (%)	NAPA binding affinity (%)	Binding affinity difference	RNA-seq in RKO (FPKM)	Knockout mouse model	Gene knockout in HAP1
AAK1	45	75	55	25	30	0.85	+	+
ABL2	14	43	86	57	29	15.13	+	+
AURKA	53	90	47	10	37	47.15		
CAMK2A	59	90	41	10	31	0.00	+	+
CDK7	49	87	51	13	38	28.01		
CDKL2	42	70	58	30	28	0.07	+	+
CLK1	25	54	75	46	29	9.16	+	+
CLK2	27	80	73	20	53	12.64	+	+
CLK4	27	64	73	36	37	3.23	+	+
CSF1R	3.9	46	96.1	54	42.1	0.03		+
CSNK2A2	29	68	71	32	39	18.84	+	+
DYRK1A	7.8	93	92.2	7	85.2	8.42		
DYRK2	63	100	37	0	37	12.80	+	+
EIF2AK4	38	83	62	17	45	23.85		+
FLT1	36	91	64	9	55	0.17		+
FLT4	35	81	65	19	46	0.00		+
GRK4	15	66	85	34	51	0.62	+	+
GSG2	6.9	40	93.1	60	33.1	14.74	+	+
HIPK1	12	52	88	48	40	9.62	+	+
HIPK2	22	72	78	28	50	5.41	+	+
HIPK3	42	93	58	7	51	8.55	+	+
HIPK4	10	66	90	34	56	0.22		+
IRAK3	46	83	54	17	37	0.00	+	+
JAK3_JH1	19	87	81	13	68	1.15	+	+
KDR	7.1	93	92,9	7	85.9	0.00		+
MAP3K9	8.5	73	91,5	27	64.5	2.78	+	+
MAP3K11	22	63	78	37	41	17.07	+	+
MAST1	46	82	54	18	36	0.78	+	+
MINK1	24	62	76	38	38	23.45	+	+
MKNK1	37	71	63	29	34	10.28	+	+
MKNK2	26	99	74	1	73	86.86	+	+
NEK10	37	69	63	31	32	0.13		+
NTRK2	6,8	54	93.2	46	47.2	0.00	+	+
NTRK3	11	61	89	39	50	0.01		+
PDGFRA	3.4	69	96.6	31	65.6	0.00		+
PIP4K2B	20	72	80	28	52	17.27	+	+
PIP5K1A	52	87	48	13	35	64.64	+	+
RET	24	73	76	27	49	0.02		+

RIOK3	21	61	79	39	40	4.12	+	+
RPS6KA1_Dom1	56	91	44	9	35	50.79	+	+
RPS6KA3_Dom1	39	67	61	33	28	4.50	+	+
RPS6KA6_Dom1	58	91	42	9	33	0.03	+	+
TNIK	39	65	61	35	26	4.49	+	+
ZAK	22	62	78	38	40	15.62	+	+

Supplementary Table 4. Kinases with the highest differential APA-NAPA binding affinity in the scanMAX Kinase Assay Panel

The amount of recovered kinase in presence of a compound normalized to the kinase capture in absence of the compound is reported as percentage of control (POC). The binding affinity of a compound towards a kinase is interpreted as 100-POC and the difference between the binding affinity of APA and NAPA is shown in the column 6. The mean of three RNA sequencing reaction on the RKO transcriptome is represented in Fragments Per Kilobase of transcript per Million mapped reads (FPKM). The availability of knock-out mouse model and/or of gene knock-out in HAP1 cell line is denoted by “+”.

Compound	$T_{1/2}$ (min)	Solubility in PBS (µM)	$f_{u,plasma}$ (%)
**APA**	12	7.6	14
**NAPA**	1.3	1.1	11

Supplementary Table 5. ADME profiling of APA and NAPA

Metabolic stability was determined in CD-1 mouse liver microsomes and is shown as in vitro half-life ($T_{1/2}$). Kinetic solubility was measured in potassium phosphate buffer. Plasma protein binding was assessed in CD-1 mouse plasma and is presented as fraction unbound in plasma ($f_{u,plasma}$).

Sample	Nucleotide change							Predicted acetylator phenotype	APA scoring
	G191A	C282T	T341C	C481T	G590A	A803G	G857A		
16-39	GG	CC	TT	CT	GA	GG	GG	Intermediate (0.612)	Resistant
16-56	GG	CC	TT	CC	GG	AA	GG	Rapid (0.980)	Resistant
16-152	GG	CT	TC	CT	GA	GA	GG	Slow (0.999)	Resistant
17-25	GG	CC	CC	TT	GG	GG	GG	Slow (0.997)	Sensitive
17-30	GG	TT	TT	CC	AA	AA	GG	Slow (0.997)	Sensitive
17-89	GG	CC	CC	TT	GG	GG	GG	Slow (0.997)	Sensitive
17-96	GG	CT	TC	CT	GA	GA	GG	Slow (0.999)	Sensitive
17-127	GG	CC	TC	CT	GG	GA	GG	Intermediate (0.997)	Sensitive
17-145	GG	CC	TC	CT	GG	GA	GG	Intermediate (0.997)	Sensitive
17-168	GG	TT	TT	CC	AA	AA	GG	Slow (0.997)	Sensitive
550	GG	CT	TT	CC	GG	AA	GG	Rapid (0.921)	Resistant
544	GG	CC	CC	TT	GG	GG	GG	Slow (0.997)	Sensitive

Supplementary Table 6. Patient tumors sensitive to APA encode NAT2 slow acetylator phenotypes

Twelve CRC primary tumors showing in vitro resistance or sensitivity towards APA (Figure 4C) were genotyped for the main SNVs that determine the NAT2 acetylator phenotype. Acetylator phenotypes were predicted with NAT2PRED [http://nat2pred.rit.albany.edu/], with a final prediction score representing the confidence in the predicted phenotype shown in parenthesis.

Gene	SNV	Forward primer (5’-3’)	Reverse primer (5’-3’)	Product size (bp)
NAT2	rs1799930	CCTGGACCAAATCAGGAGAG	GATGAAGCCCACCAAACAGT	235
	rs1801280	GCTCTGACCACAATCGGTTT	GGCTGATCCTTCCCAGAAAT	200
ABP1	rs1049793	CCTGGCAGTGACCAAGTACC	TCCCTGATGGTGATGAGGAT	211
AKR7A2	rs1043657	AACCCTTGGGATGGAAAATC	CTGGTGCCTCTGCTCTCAT	250
SULT1C3	rs2219078	CCTTGGGTTTTGTCCCAGTA	TTGGCCAATTAAAGACACAGC	234
SULT1A1	rs9282861	TCAGTAATCCGAGCCTCCAC	GCTGTGGTCCATGAACTCCT	201

Supplementary Table 7. Primer pairs used for PCR amplification of SNVs of interest
