# Supplementary material for: Exploiting loss of heterozygosity for allele-selective colorectal cancer chemotherapy
Source: Nat Commun. 2020 Mar 11;11:1308. doi: 10.1038/s41467-020-15111-4 (PMC7066191; doi:10.1038/s41467-020-15111-4)
Supplement: Supplementary file 2 — Description of Additional Supplementary Information [file 41467_2020_15111_MOESM2_ESM.pdf]

Description of Additional Supplementary Files

File Name: Supplementary Data 1
Description: Prevalent SNVs that alter amino acids in or near active surfaces of intracellular enzymes
