# Supplementary material for: Exploiting loss of heterozygosity for allele-selective colorectal cancer chemotherapy
Source: Nat Commun. 2020 Mar 11;11:1308. doi: 10.1038/s41467-020-15111-4 (PMC7066191; doi:10.1038/s41467-020-15111-4)
Supplement: Supplementary file 4 — Reporting Summary [file 41467_2020_15111_MOESM4_ESM.pdf]

nature research

Corresponding author(s): Prof. Tobias Sjöblom
NCOMMS-18-15793B-Z

Last updated by author(s): Jan 6, 2020

Reporting Summary

Nature Research wishes to improve the reproducibility of the work that we publish. This form provides structure for consistency and transparency in reporting. For further information on Nature Research policies, see Authors & Referees and the Editorial Policy Checklist .

Statistics

For all statistical analyses, confirm that the following items are present in the figure legend, table legend, main text, or Methods section.

n/a	Confirmed	
☐	☒	The exact sample size (*n*) for each experimental group/condition, given as a discrete number and unit of measurement
☐	☒	A statement on whether measurements were taken from distinct samples or whether the same sample was measured repeatedly
☐	☒	The statistical test(s) used AND whether they are one- or two-sided<br>*Only common tests should be described solely by name; describe more complex techniques in the Methods section.*
☒	☐	A description of all covariates tested
☒	☐	A description of any assumptions or corrections, such as tests of normality and adjustment for multiple comparisons
☐	☒	A full description of the statistical parameters including central tendency (e.g. means) or other basic estimates (e.g. regression coefficient) AND variation (e.g. standard deviation) or associated estimates of uncertainty (e.g. confidence intervals)
☐	☒	For null hypothesis testing, the test statistic (e.g. *F*, *t*, *r*) with confidence intervals, effect sizes, degrees of freedom and *P* value noted<br>*Give P values as exact values whenever suitable.*
☒	☐	For Bayesian analysis, information on the choice of priors and Markov chain Monte Carlo settings
☒	☐	For hierarchical and complex designs, identification of the appropriate level for tests and full reporting of outcomes
☒	☐	Estimates of effect sizes (e.g. Cohen's *d*, Pearson's *r*), indicating how they were calculated

Our web collection on statistics for biologists contains articles on many of the points above.

Software and code

Policy information about availability of computer code

Data collection	RT-qPCR analysis: Data were analyzed by the ΔΔCt method using the StepOne™ Software v2.1 (Thermo Fisher Scientific).<br>Immunofluorescence stainings: Imaging was done in a Leica SP8 confocal microscope using the Leica application suite X fluorescence software. Image acquisition was done with a 63× objective, followed by processing and quantification with ImageJ (NIH) and CellProfiler (Broad Institute).<br>Western Blots: The immunoblot quantification was performed by ImageJ software (NIH) and the data for normalization to the loading control were obtained following integration of the band intensity.
Data analysis	No proprietary algorithms were used and all steps can be reproduced by using publicly available datasets and the filtering criteria in Figure 1A.

For manuscripts utilizing custom algorithms or software that are central to the research but not yet described in published literature, software must be made available to editors/reviewers. We strongly encourage code deposition in a community repository (e.g. GitHub). See the Nature Research guidelines for submitting code & software for further information.

Data

Policy information about availability of data

All manuscripts must include a data availability statement. This statement should provide the following information, where applicable:

- Accession codes, unique identifiers, or web links for publicly available datasets
- A list of figures that have associated raw data
- A description of any restrictions on data availability

Publicly available datasets:
Data for human genetic variation were obtained from Phase 1 samples of the 1000 Genomes project (Release 3, 2012-04-30). The consensus coding DNA sequence definitions of human protein coding exons were retrieved from the CCDS database (Release 15, 2013-11-29). To map the remaining SNVs to functional protein domains, we translated genome coordinates into protein coordinates using the UniProtKB database (Release 2013_12). The resulting set of SNVs was enriched for

potential relevance to catalytic activity by matching to the functional domain definitions in the Pfam 27.0 database (Release 2013-03-15).

The authors declare that the data supporting the findings of this study are available within the paper and its supplementary information files.

Field-specific reporting

Please select the one below that is the best fit for your research. If you are not sure, read the appropriate sections before making your selection.

☒ Life sciences ☐ Behavioural & social sciences ☐ Ecological, evolutionary & environmental sciences

For a reference copy of the document with all sections, see nature.com/documents/nr-reporting-summary-flat.pdf

Life sciences study design

All studies must disclose on these points even when the disclosure is negative.

Sample size

In vitro experiments:
All experiments were conducted in at least two replicates and were reported as the mean of at least two different biological repeats.

In vivo experiments:
Statistical power analysis was performed to determine the minimum number of animals per experimental arm required to demonstrate a statistically significant difference in treatment outcomes. The model adopted was the independent t-test for 2 samples as follows.
n = [(s1^2 + s2^2)*(z_crit * z_pwr)^2]/D^2
where,
n = size of sample group
s1 = standard deviation of control group
s2 = standard deviation of sample group
z_crit = 1.960 (standard normal deviate for significance criterion 0.05; CI = 95%)
z_pwr = 0.842 (standard normal deviate for statistical power 0.80)
The observable tumor volume range lies between 0 to 2000 mm^3. Our aim is to observe a minimum difference of 100 mm^3 (i.e. D = 100) between the control and sample group mean volumes with statistical significance. The power of our study is 0.8 and the probability of a type I error is nominally taken to be 0.05. From historical data, we have estimated the standard deviation within each group to be 80 mm^3 (i.e. s1 = 80 and s2 = 80). Based on these estimates:
n = [(802 + 802) * (1.960 * 0.842)^2]/100^2 = 10 mice per group

Data exclusions

No data were excluded from the analyses.

Replication

In vitro assays were performed in at least three independent biological replicates to achieve reproducibility of the results. Data are presented as arithmetic mean of all replicates with standard deviations.

Randomization

Described in section "APA treatment of mouse xenograft models" of Methods, where animals were randomly assigned to each treatment arm.

Blinding

The tumour-bearing mice were subjected to treatment in a non-blinded experiment.

Reporting for specific materials, systems and methods

We require information from authors about some types of materials, experimental systems and methods used in many studies. Here, indicate whether each material, system or method listed is relevant to your study. If you are not sure if a list item applies to your research, read the appropriate section before selecting a response.

Materials & experimental systems

n/a	Involved in the study	
☐	☒	Antibodies
☐	☒	Eukaryotic cell lines
☒	☐	Palaeontology
☐	☒	Animals and other organisms
☐	☒	Human research participants
☒	☐	Clinical data

Methods

n/a	Involved in the study	
☒	☐	ChIP-seq
☒	☐	Flow cytometry
☒	☐	MRI-based neuroimaging

Antibodies

Antibodies used

Primary antibodies:
rabbit α-Myc (71D10, Cell Signaling Technology)

mouse α-Tubulin (T8203, Sigma Aldrich)
mouse α-Tubulin (T9026, Sigma-Aldrich)
rabbit anti-phospho-Aurora A (C39D8, Cell Signaling Technology)
rabbit anti-phospho-histone H3 (pSer10) (04-817, Merck)
rabbit anti-Aurora A (ab52973, Abcam)
mouse anti-PCNA (PC10, sc-56, Santa Cruz Biotechnology)
rabbit polyclonal anti-histone H3 (ab1791, Abcam)

Secondary antibodies:
goat anti-rabbit (31460, Thermo Scientific)
goat anti-mouse (31430, Thermo Scientific)
Alexa-488 donkey anti-mouse (A21202, Invitrogen)
Alexa-467 donkey anti-rabbit (A31573, Invitrogen)

Validation

All antibodies were provided by commercial vendors with respective source and product numbers stated in the text. All antibodies were certified by the provider to work for analysis of human proteins for the intended assay.

Product citations (n):
rabbit α-Myc (71D10, Cell Signaling Technology), n = 249 (https://www.cellsignal.com/products/primary-antibodies/myc-tag-71d10-rabbit-mab/2278)
mouse α-Tubulin (T8203, Sigma Aldrich), n = 116 (https://www.sigmaaldrich.com/catalog/product/sigma/t8203?lang=en&region=US)
mouse α-Tubulin (T9026, Sigma-Aldrich), n = 2190 (https://www.sigmaaldrich.com/catalog/product/sigma/t9026?lang=en&region=US)
rabbit anti-phospho-Aurora A (C39D8, Cell Signaling Technology), n = 54 (https://www.cellsignal.com/products/primary-antibodies/phospho-aurora-a-thr288-c39d8-rabbit-mab/3079)
rabbit anti-phospho-histone H3 (pSer10) (04-817, Merck), n = 13 (https://www.emdmillipore.com/US/en/product/Anti-phospho-Histone-H3-Ser10-Antibody-clone-MC463-rabbit-monoclonal,MM_NF-04-817)
rabbit anti-Aurora A (ab52973, Abcam), n = 3 (https://www.abcam.com/aurora-a-antibody-ep1008y-ab52973.html)
mouse anti-PCNA (PC10, sc-56, Santa Cruz Biotechnology), n = 2124 (https://www.scbt.com/p/pcna-antibody-pc10)
rabbit polyclonal anti-histone H3 (ab1791, Abcam), n = 2897 (https://www.abcam.com/histone-h3-antibody-nuclear-loading-control-and-chip-grade-ab1791.html)
goat anti-rabbit (31460, Thermo Scientific), n = 67 (https://www.thermofisher.com/antibody/product/Goat-anti-Rabbit-IgG-H-L-Secondary-Antibody-Polyclonal/31460)
goat anti-mouse (31430, Thermo Scientific), n = 70 (https://www.thermofisher.com/antibody/product/Goat-anti-Mouse-IgG-H-L-Secondary-Antibody-Polyclonal/31430)
Alexa-488 donkey anti-mouse (A21202, Invitrogen), n = 68 (https://www.thermofisher.com/antibody/product/Donkey-anti-Mouse-IgG-H-L-Highly-Cross-Adsorbed-Secondary-Antibody-Polyclonal/A-21202)
Alexa-467 donkey anti-rabbit (A31573, Invitrogen) , n = 22 (https://www.thermofisher.com/antibody/product/Donkey-anti-Rabbit-IgG-H-L-Highly-Cross-Adsorbed-Secondary-Antibody-Polyclonal/A-31573)

Eukaryotic cell lines

Policy information about cell lines

Cell line source(s)

The human colorectal cancer cell lines RKO, DLD-1 and HCT116 were commercially acquired from ATCC.

Authentication

The cell lines were authenticated by STR profiling (ATCC cell authentication service).

Mycoplasma contamination

All cell lines were regularly checked for mycoplasma infection with the MycoAlert mycoplasma detection kit (Lonza). All cell lines in this study tested negative for mycoplasma contamination.

Commonly misidentified lines
(See ICLAC register)

The cell lines used in this paper are not listed in the database of commonly misidentified cell lines (ICLAC).

Animals and other organisms

Policy information about studies involving animals; ARRIVE guidelines recommended for reporting animal research

Laboratory animals

Six week old BALB/c nude athymic mice (female) were used in this study's in vivo experiments. Mice were housed at 25 C and exposed to a 12-hour dark/light regime. All animals were fed ad libitum.

Wild animals

No wild animals were used in this study.

Field-collected samples

No field-collected samples were used in this study.

Ethics oversight

All animal experiments were overseen and approved by the Institutional Animal Care and Use Committee of Temasek Life Sciences Laboratory at the National University of Singapore (#TLL-14-022).

Note that full information on the approval of the study protocol must also be provided in the manuscript.

Human research participants

Policy information about studies involving human research participants

Population characteristics

The NAT2 genotyping cohort comprises of 74 individuals with colorectal cancer (CRC) with representation of disease stages II, III and IV. Tumor samples were obtained from CRC patients operated for peritoneal metastasis. The patient sampling was approved by the regional ethical committee, Uppsala University (file Dnr 2007/237).

Organoid samples from CRC patients were obtained after approval from the Gastrointestinal Cancer Center at the Dana-Farber Cancer Institute (protocol numbers 03-189, 17-000 and 18-060).

No data describing age, gender or other genetic information was obtained for any of the genotyped individuals.

Recruitment

Patient samples were recruited based on availability without biases towards gender or disease stage. The cohort was used merely to assess the NAT2 genotype in a CRC population.

Ethics oversight

All human research studies were conducted after participation consent and approval from the ethical committees at Uppsala University (Sweden) and Dana-Farber Cancer Institute (USA).

Note that full information on the approval of the study protocol must also be provided in the manuscript.
