# Supplementary figures and images for: Exploiting loss of heterozygosity for allele-selective colorectal cancer chemotherapy
Source: Nat Commun. 2020 Mar 11;11:1308. doi: 10.1038/s41467-020-15111-4 (PMC7066191; doi:10.1038/s41467-020-15111-4)

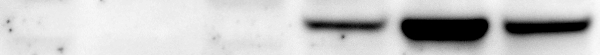

Supplement: Supplementary file 5 — Source Data [file 41467_2020_15111_MOESM5_ESM.zip › Figure_2AB/DLD_Myc/DLD-cMyc_1min.jpg]

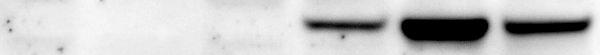

Supplement: Supplementary file 5 — Source Data [file 41467_2020_15111_MOESM5_ESM.zip › Figure_2AB/DLD_Myc/DLD-cMyc_1min.tif]

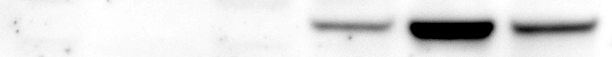

Supplement: Supplementary file 5 — Source Data [file 41467_2020_15111_MOESM5_ESM.zip › Figure_2AB/DLD_Myc/DLD-cMyc_30sek.jpg]

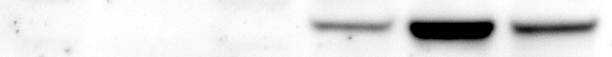

Supplement: Supplementary file 5 — Source Data [file 41467_2020_15111_MOESM5_ESM.zip › Figure_2AB/DLD_Myc/DLD-cMyc_30sek.tif]

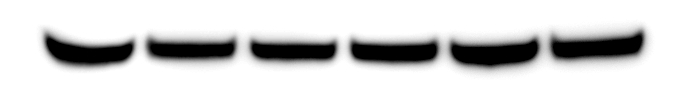

Supplement: Supplementary file 5 — Source Data [file 41467_2020_15111_MOESM5_ESM.zip › Figure_2AB/DLD_Myc/DLD-cMyc_TubulinLoading_2sek.jpg]

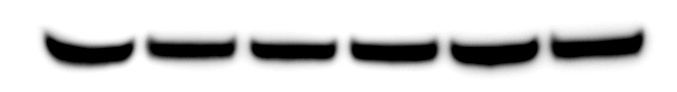

Supplement: Supplementary file 5 — Source Data [file 41467_2020_15111_MOESM5_ESM.zip › Figure_2AB/DLD_Myc/DLD-cMyc_TubulinLoading_2sek.tif]

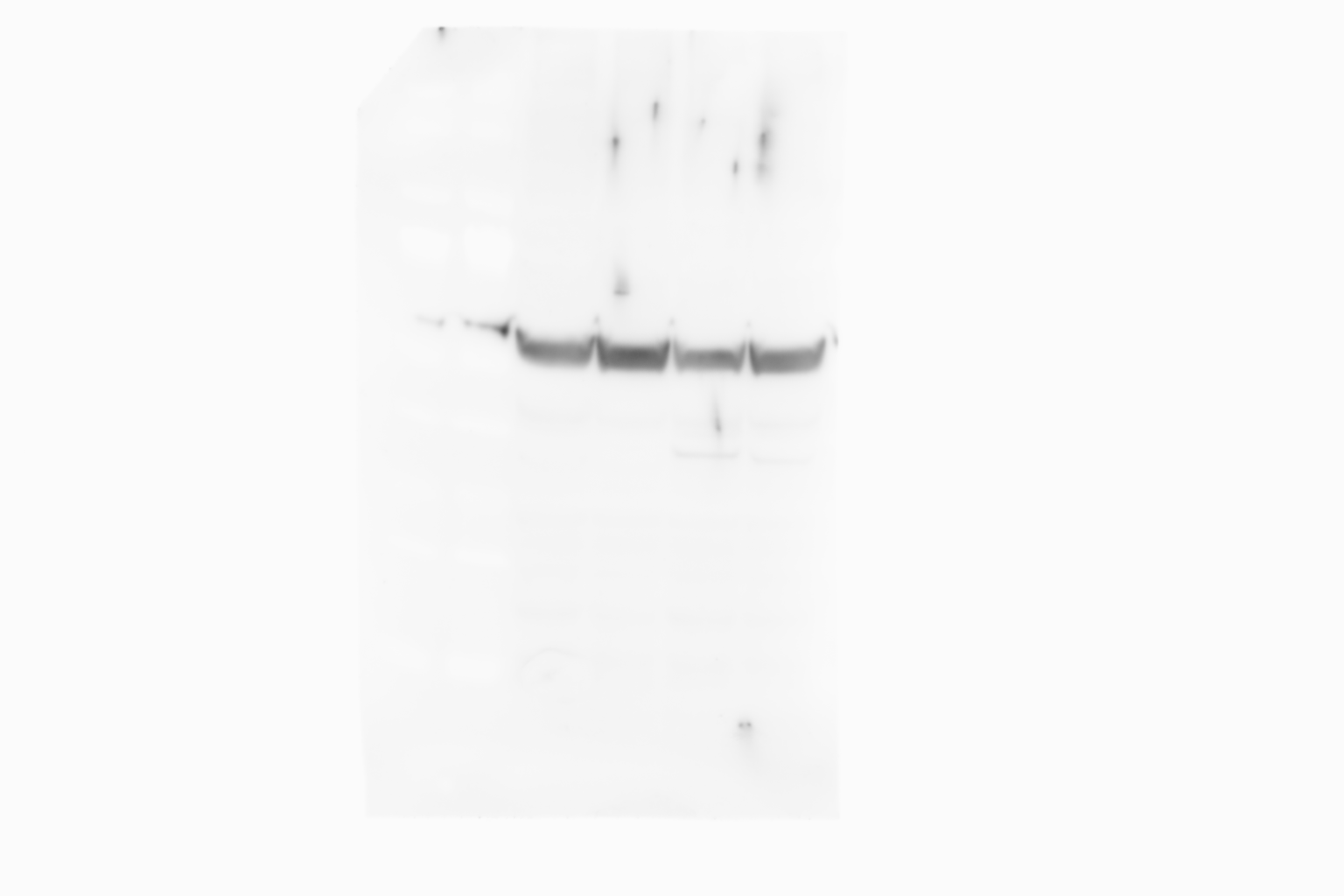

Supplement: Supplementary file 5 — Source Data [file 41467_2020_15111_MOESM5_ESM.zip › Figure_2AB/RKO_Myc/Loading_DayAfter_30sekprec.tif]
